# Endothelial progenitor cells derived from embryonic stem cells prevent alveolar simplification in a murine model of bronchopulmonary dysplasia

**DOI:** 10.3389/fcell.2023.1209518

**Published:** 2023-06-09

**Authors:** Olena A. Kolesnichenko, Hannah M. Flood, Yufang Zhang, Vladimir Ustiyan, Hayde K. Cuervo Jimenez, Tanya V. Kalin, Vladimir V. Kalinichenko

**Affiliations:** ^1^ Center for Lung Regenerative Medicine, Cincinnati Children’s Hospital Medical Center, Cincinnati, OH, United States; ^2^ Division of Pulmonary Medicine, Cincinnati Children’s Hospital Medical Center, Cincinnati, OH, United States; ^3^ Division of Pulmonary Biology, Cincinnati Children’s Hospital Medical Center, Cincinnati, OH, United States; ^4^ Phoenix Children’s Health Research Institute, Department of Child Health, University of Arizona College of Medicine—Phoenix, Phoenix, AZ, United States; ^5^ Division of Neonatology, Phoenix Children’s Hospital, Phoenix, AZ, United States

**Keywords:** endothelial progenitor cells, directed differentiation of embryonic stem cells, bronchopulmonary dysplasia, cellular therapy, FOXF1 gene

## Abstract

**Introduction:** Vascular remodeling and compromised alveolar development are hallmarks of chronic pulmonary diseases such as bronchopulmonary dysplasia (BPD). Despite advances in neonatal healthcare the number of BPD cases worldwide continues to increase. One approach to overcoming the premature arrest in lung development seen in BPD is to stimulate neonatal angiogenesis via delivery and engraftment of endothelial progenitor cells (EPCs). One such population is resident to the pulmonary microvasculature and expresses both FOXF1 and c-KIT. Previous studies have shown that c-KIT^+^FOXF1^+^ EPCs are highly sensitive to elevated levels of oxygen (hyperoxia) and are decreased in premature infants with BPD and hyperoxia-induced BPD mouse models. We hypothesize that restoring EPCs through transplantation of c-KIT^+^FOXF1^+^ EPCs derived *in vitro* from pluripotent embryonic stem cells (ESCs), will stimulate neonatal angiogenesis and alveolarization in mice with hyperoxia-induced lung injury.

**Methods:** Utilizing a novel ESC line with a FOXF1:GFP reporter, we generated ESC-derived c-KIT^+^FOXF1^+^ EPCs *in vitro*. Using a second ESC line which contains FOXF1:GFP and tdTomato transgenes, we differentiated ESCs towards c-KIT^+^FOXF1^+^ EPCs and tracked them *in vivo* after injection into the neonatal circulation of hyperoxia-injured mice. After a recovery period in room air conditions, we analyzed c-KIT^+^FOXF1^+^ EPC engraftment and quantified the number of resident and circulating endothelial cells, the size of alveolar spaces, and the capillary density after EPC transplantations.

**Results and conclusion:** Herein, we demonstrate that addition of BMP9 to the directed endothelial differentiation protocol results in very efficient generation of c-KIT^+^FOXF1^+^ EPCs from pluripotent ESCs. ESC-derived c-KIT^+^FOXF1^+^ EPCs effectively engraft into the pulmonary microvasculature of hyperoxia-injured mice, promote vascular remodeling in alveoli, increase the number of resident and circulating endothelial cells, and improve alveolarization. Altogether, these results provide a proof-of-principle that cell therapy with ESC-derived c-KIT^+^FOXF1^+^ EPCs can prevent alveolar simplification in a hyperoxia-induced BPD mouse model.

## Introduction

Bronchopulmonary dysplasia (BPD) is a multifactorial, developmental lung disease affecting low birth weight and premature infants, especially those born before 28 weeks gestation (Davidson and Berkelhamer, 2017; Mowitz et al., 2019). The hallmarks of BPD include alveolar simplification, respiratory insufficiency requiring supplemental oxygen, inflammation, and variable fibrotic remodeling (Stenmark and Abman, 2005). Vascular abnormalities are observed in severe cases of BPD and are associated with increased morbidity and mortality (Alvira, 2016; Thebaud et al., 2019). Since its first description in 1967 (Northway et al., 1967), the characteristics of BPD have evolved, mirroring the advances in neonatal medicine. However, despite new treatments such as surfactant therapy, steroid administration, and improved ventilation strategies, the number of BPD cases continues to increase.

Angiogenesis and vasculogenesis are two distinct biological processes which result in the formation of new blood vessels in the lung. Angiogenesis occurs when new vessels sprout from existing vessels. Conversely, vasculogenesis describes the *de novo* formation of blood vessels from differentiating endothelial progenitor cells (EPCs). In the developing embryo, vasculogenesis occurs in extraembryonic tissues where EPCs undergo a stepwise differentiation from mesoderm progenitors to hemangioblasts and angioblasts, ultimately giving rise to blood islands which fuse to form the primitive vascular plexus (Kolesnichenko et al., 2021). Subsequent rapid growth and sprouting of pulmonary endothelium, results in the formation of the complex vascular network containing general capillary cells (gCaps or CAP1), aerocytes (aCaps or CAP2), arterial, venous, and lymphatic endothelial cells (Whitsett et al., 2019; Gillich et al., 2020). Vascular remodeling of the mature lung was believed to occur only by means of angiogenesis until 1997, when Asahara and others published their seminal findings describing adult putative EPCs (Asahara et al., 1997). Since then, various circulating and tissue resident EPC populations have been described (Liu et al., 2015; Gillich et al., 2020; Niethamer et al., 2020; Vila Ellis et al., 2020). One such population which resides in the lung microvasculature is identified by its dual cell surface expression of c-KIT and FOXF1 (Ren et al., 2019).

Forkhead box F1 (FOXF1) is an evolutionarily conserved transcription factor required for embryonic development (Dharmadhikari et al., 2015). *Foxf1* ^*−/−*^ mice are embryonic lethal due to the lack of vascular development in the yolk sac and allantois (Ren et al., 2014). Heterozygous deletion of *Foxf1* or the S52F mutation in mice result in increased morbidity and mortality due to lung hypoplasia and impaired pulmonary microvascular development (Pradhan et al., 2019). Loss of gene locus and point mutations in human *FOXF1* have been described in patients with Alveolar capillary dysplasia with misalignment of pulmonary veins (ACDMPV), a rare congenital disorder resulting in decreased alveolar capillary density, abnormal positioning of pulmonary veins, and respiratory insufficiency in infants after birth (Bishop et al., 2011; Towe et al., 2018). Recent single-cell RNA sequencing of mouse and human lungs showed that FOXF1, along with its transcriptional target c-KIT (Ren et al., 2019), are expressed in a population of lung-resident EPCs. C-KIT^+^FOXF1^+^ EPCs are highly sensitive to increased levels of oxygen (hyperoxia) (Ren et al., 2019) and are shown to be decreased in premature infants which develop BPD as well as in hyperoxia-induced BPD mouse models (Borghesi et al., 2009; Fujinaga et al., 2009). Adoptive transfer of c-KIT^+^ EPCs into systemic circulation of hyperoxia-injured mice results in the engraftment of EPCs into the alveolar microvasculature, increased neonatal lung angiogenesis and improved alveolarization in the mouse BPD model (Ren et al., 2019). This cellular therapy, although effective, relies on the availability of donor lung tissue. Additionally, the cell number obtained for the therapy is limited by the harsh method of cell sorting, required to obtain EPCs.

In the present study, we generated a novel murine embryonic stem cell (ESC) line with a GFP reporter knocked into the endogenous *Foxf1* gene locus and used these cells to produce c-KIT^+^FOXF1^+^ EPCs via directed differentiation of ESCs *in vitro*. We then introduced ESC-derived EPCs into the circulation of hyperoxia-injured BPD mice and demonstrated that cellular therapy with *in vitro* differentiated c-KIT^+^FOXF1^+^ EPCs can increase neonatal lung angiogenesis and decrease alveolar simplification in a murine model of BPD.

## Materials and methods

### Generation of embryonic stem cell lines

The W4 parental embryonic stem cell (ESC) line, derived from 129S6 mice, was generated and described previously (Auerbach et al., 2000). The A1 ESC line with a GFP reporter knocked in to the *Foxf1* locus (FOXF1:GFP) cell line was generated from the W4 parental line by the Transgenic Animal and Genome Editing Core at Cincinnati Children’s Hospital Medical Center. Briefly, the FOXF1:GFP (Supplementary Figure S1A) and CRISPR plasmid constructs were introduced into the parental, W4 ESCs using electroporation. GFP positive cells were sorted into 96-well plates. Thirty GFP positive clones were recovered after plating. PCR screening further confirmed 6 positive clones (Supplementary Figure S1B); three homozygous (A1, D2, and G1) and 3 heterozygous clones (C2, E4, and G2). The WT and non-GFP allele sequences of the heterozygous clones are provided in Supplementary Table S1. The FOXF1:GFP/tdTomato ESC line (C57Bl/6 × 129J x CD1) was generated, karyotyped and described previously (Wang et al., 2021).

### Differentiation of embryonic stem cells to endothelial progenitor cells

Embryonic stem cell lines were seeded on Matrigel (Corning Life Sciences)-coated surfaces and maintained in 2i media, supplemented with MEK inhibitor PD0325901, GSK-3 inhibitor CHIR99021 (Stemgent) and leukemic inhibitory factor, LIF (Sigma-Aldrich). ESCs were maintained and passaged every 2–3 days as needed, based on cell density. Differentiation consisted of two stages, 1) priming and 2) differentiation. All growth factors applied to both the priming and differentiation medias were fist dissolved in the manufacturer’s recommended solvent at 0.1 mg/mL then added to the culture media at 1:10,000 (μL). Prior to differentiation, day −1, ESCs were singularized using 0.05% Trypsin EDTA (Gibco) and seeded in Matrigel-coated 6-well plates at a density of approximately 5.0 × 10^4^ cells/well. To initiate differentiation at day 0, cells were treated with priming media consisting of a -2i media base (2i media without the inhibitors and LIF) supplemented with Activin A (R&D Systems, 338-AC-010/CF), BMP4 (R&D Systems, 5020-BP-010/CF), and EGF (Gold Biotechnology, 1350-04-500). After 24 h, the medium was changed to differentiation media consisting of -2i supplemented with EGF, FGF2 (Gold Biotechnology, 0340-02-50), SHH (Gold Biotechnology, 6310-19-5) or BMP9 (R&D Systems, 5566-BP-010/CF), and VEGF_165_ (Gold Biotechnology, 1350-07-10). Fresh differentiation media was applied to the cells as needed. After day 5, cells were either collected for further analysis or passaged and maintained as needed.

### Flow cytometry

Flow cytometry was performed on single cell suspensions from enzyme-digested whole lungs as described previously (Sun et al., 2017; Wang et al., 2021) or cells obtained after ESC differentiation towards EPCs *in vitro*. Cultured cells were dissociated with Dispase (StemCell), washed with PBS, filtered through a 70 um filter and counted to obtain the final cell count of the single cell suspension. Live cells were identified with 7-aminoactinomycin D (7-AAD) (BioLegend). Hematopoietic cells were identified using CD45 antibody (Thermofisher; 56-0451-82). The CD45^−^ population was then evaluated for expression of CD31 (R&D; AF3528), GFP (from FOXF1:GFP transgene), and c-KIT (CD117; eBiosceince; 78-1171-82). Endothelial cells were identified as CD31^+^, CD45^−^. Differentiated embryonic stem cells (ESCs) were identified using tdTomato transgene and c-KIT^+^FOXF1^+^ EPCs (tdTomato^+^, GFP^+^, c-KIT^+^) cells were FACS-sorted using the FACSAria II, five-laser cell sorter (BD Biosciences).

### Neonatal hyperoxia and EPC injection

For these studies wild-type (WT) C57BL/6 mice were used. Two hyperoxia regimens were used to induce the BPD phenotype in newborn mice. In the first regimen, newborn mice were exposed to 75% O_2_ for 7 days (postnatal days (P) P1-7). At P7, pups were removed from hyperoxia chambers and placed in room air approximately 2 h prior to EPC injection. Cells were injected retro orbitally and mice were left to recover in a room air environment. In the second regimen, newborn mice were exposed to 85% O_2_ for 5 days (P1-P5). Mice were placed in room air prior to and post-injection, as described above. In both regimens, mice were injected retro orbitally and the concentrations of injected cells were 60,000/mouse in regimen 1, and 100,000 and 400,000/mouse in regimen 2.

### Histology and immunostaining

Frozen lung sections were stained with hematoxylin and eosin (H&E) or used for immunostaining as previously described (Kalinichenko et al., 2003; Kim et al., 2005; Ustiyan et al., 2018) with the following antibodies (Abs): NANOG (1:1,000; Cell Signaling; #8822), SOX2 (1:200; Seven Hills; WRAB-1236), SOX9 (1:250; MilliporeSigma; AB5535), Endomucin (1:100; Abcam; ab106100), PECAM-1 (1:200; R&D Systems; AF3628; 1:200; Abcam; ab28364), TTF1 (1:250; Seven Hills; WRAB-1231), FOXF1 (1:100; R&D Systems; AF4798), Ki-67 (1:250; BD Bioscience; #550609), Cleaved Caspase-3 (1:100; Cell Signaling; #9664), aSMA (1:2,000; Sigma-Aldrich; A5228), NG2 (1:100; EMD Millipore; AB5320A4), GPIHBPI (1:300; Thermofisher Scientific; PA5-16976), CAR4 (1:250; R&D Systems; AF2414). Changes in alveolar size were determined as previously described (Xia et al., 2015) using the morphometric analysis of H&E-stained lung sections. The PECAM-1 (CD31) coverage in alveoli was determined using ImageJ quantification of CD31-stained lung sections.

### Statistical analysis

One-way ANOVA and Student’s *t*-test were used to determine statistical significance. *p* < 0.05 was considered statistically significant. All values were provided as mean ± standard deviation of the mean (SD).

## Results

### Directed differentiation of FOXF1: GFP embryonic stem cells results in the generation of a c-KIT^+^FOXF1^+^ EPC population

**FIGURE 1:**
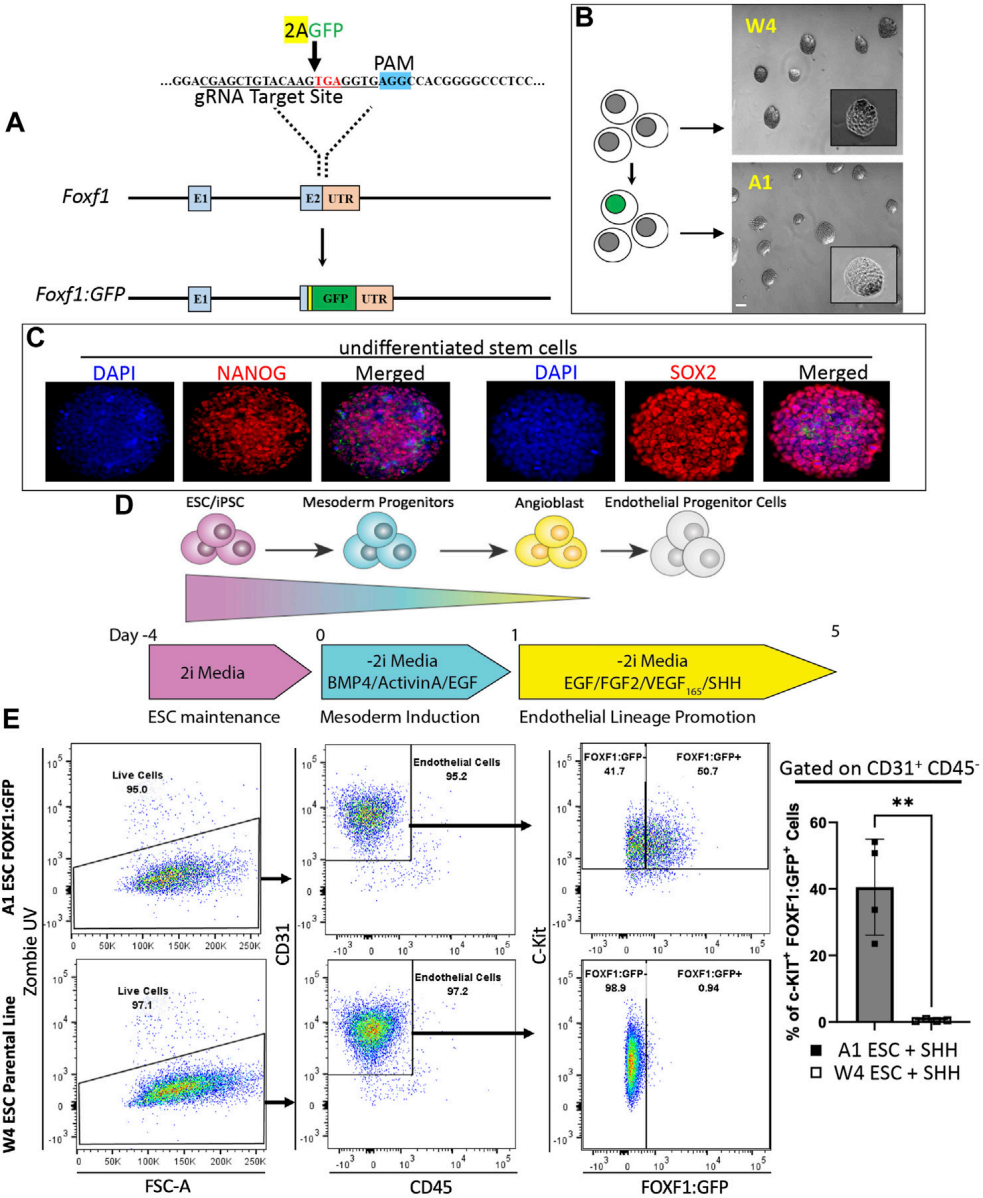
Directed differentiation of FOXF1:GFP embryonic stem cells (ESCs) results in the generation of a c-KIT^+^FOXF1^+^ endothelial progenitor cell (EPC) population. **(A)** Schematic shows the targeting strategy for CRISPR/Cas9 gene editing to introduce the AA-GFP construct into the 3′ region of the *Foxf1* gene locus without disrupting the *Foxf1* coding sequence, to generate the novel A1 ESC line. **(B)** Brightfield images show a comparison of ESC colony morphology between the parental (W4) and genetically altered FOXF1:GFP (A1) ESCs. Scale bar = 50 μm. **(C)** Immunofluorescent staining shows that A1 ESCs express appropriate ESC markers such as Nanog Homeobox (NANOG) and SRY-box transcription factor 2 (SOX2) prior to the start of directed differentiation. Image magnification is ×20. This experiment was repeated 3 times with similar results. **(D)** The schematic shows the directed differentiation protocol starting with ESC maintenance, mesoderm induction, and endothelial lineage promotion with the modification of added Sonic Hedgehog (SHH) to promote expression of FOXF1. **(E)** Flow cytometry analysis shows the result of W4 and A1 ESC differentiation. Both cell lines produce mainly endothelial cells as marked by CD31^+^CD45^−^ cell surface expression. Within the endothelial cell compartment, A1 ESCs produce a combination of c-KIT^+^FOXF1^+^ and c-KIT^+^FOXF1^-^ populations. Parental W4 ESCs lack GFP^+^ cells due to the lack of FOXF1:GFP-reporter. Values are shown as mean ± SD. This experiment was repeated 4 times, **p < 0.01.

To generate c-KIT^+^FOXF1^+^ EPCs through directed differentiation of ESCs *in vitro*, we produced a murine embryonic stem cell (ESC) line with a GFP reporter knocked into the endogenous *Foxf1* gene locus (Figure 1A). CRISPR/Cas9 gene editing was used to introduce the AA-GFP construct into the 3’ region of the *Foxf1* gene locus without disrupting the *Foxf1* coding sequence, genetically altering W4 (parental) ESCs derived from 129S6 mice (Supplementary Figure S1A). Using PCR, 30 W4 clones were assessed (Supplementary Figure S1B). Three homozygous (A1, D2, G1) and three heterozygous (C2, E4, G2) clones were identified and the latter sequenced (Supplementary Table S1). Of the homozygous clones identified, the A1 line was chosen for subsequent experiments. A1 ESC colonies grew similar to parental W4 cells as visualized by brightfield microscopy (Figure 1B). A1 ESCs expressed stem cell markers such as Nanog Homeobox (NANOG) and SRY-box transcription factor 2 (SOX2) (Figure 1C). While A1 ESCs showed low GFP expression at baseline (Supplementary Figure S1C), GFP expression increased when spontaneous differentiation was initiated with the addition of Vascular endothelial growth factor (VEGF) or Fibroblast growth factor 2 (FGF2) to the culture medium (Supplementary Figures S2A, B). As expected, the parental W4 line showed no GFP expression under these conditions (Supplementary Figures S2A–C).

Next, we modified and employed an established directed differentiation protocol to generate endothelial cells from pluripotent ESCs *in vitro* (Figure 1D) (Palpant et al., 2015; Nguyen et al., 2016; Palpant et al., 2017; Nelson et al., 2021). ESCs were maintained in 2i media and passaged 1–2 days prior to the start of differentiation. On day 0, the start of differentiation and mesoderm induction, 2i media was replaced with -2i media containing Bone morphogenetic protein 4 (BMP4), Epidermal growth factor (EGF), and Activin A (priming media). Twenty-four hours later (day 1), priming media was removed and replaced with EPC differentiation media and -2i containing EGF, FGF2, and VEGF. We also added Sonic hedgehog (SHH) at the last stage of the endothelial differentiation protocol because SHH was shown to activate transcription of *Foxf1* in the developing lung (Mahlapuu et al., 2001; Ustiyan et al., 2018). On day 5, cells were harvested and analyzed by fluorescence-activated cells sorting (FACS). FACS analysis revealed that the endothelial differentiation protocol could efficiently generate endothelial cells (CD31^+^CD45^−^) from ESCs *in vitro* (Figure 1E). Based on the FOXF1:GFP reporter and cell surface expression of c-KIT, ESC-derived endothelial cells could be subdivided into c-KIT^+^FOXF1^+^ and c-KIT^+^FOXF1^−^ populations (Figure 1E). As expected, no GFP^+^ cells were detected by flow cytometry in the parental line after undergoing the differentiation protocol (Figure 1E). Furthermore, comparison of in vitro-differentiated W4 parental and A1 FOXF1-reporter ESCs (Supplementary Figure S3C) with endogenous mouse endothelial cells, revealed similarities in expression pattern of additional endothelial markers such as CD34 and CD309 (Supplementary Figures S3A–C). CD144 was not expressed in ESC-derived endothelial cells (Supplementary Figures S3A–C). Thus, we have developed a murine ESC line capable of tracking FOXF1 expression and demonstrated that these ESCs are capable of differentiating into endothelial cells expressing FOXF1 and c-KIT.

### Transplantation of ESC-derived cells results in the formation of lung-specific cell clusters with many cell types originating from donor ESCs

**FIGURE 2:**
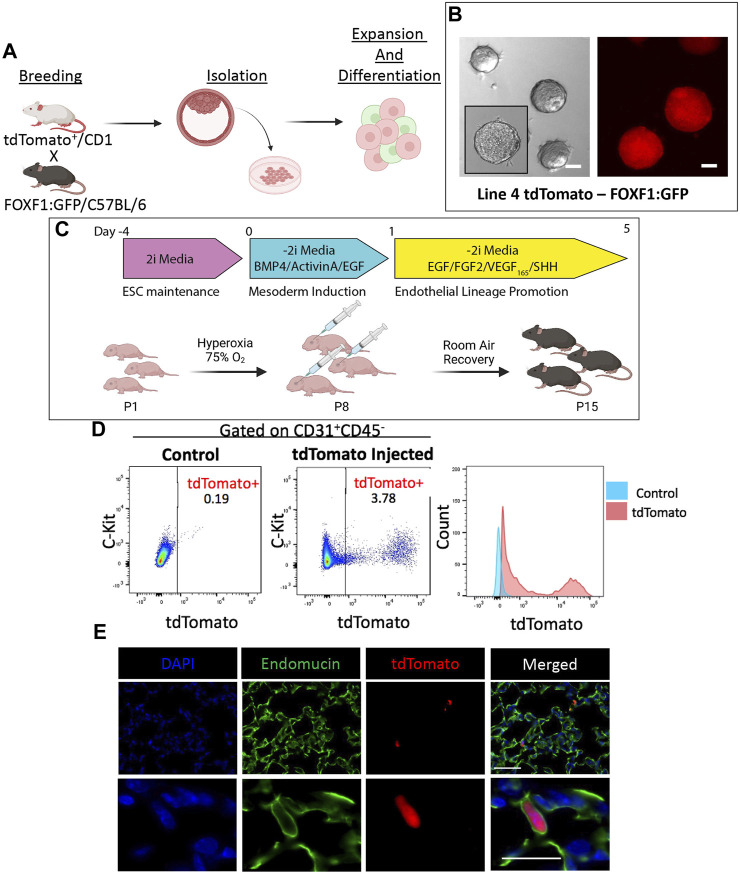
Directed differentiation and bulk injection of tdTomato^+^FOXF1: GFP ESCs results in engraftment of donor cells in lung tissue of recipient mice. **(A)** Schematic shows the process of developing tdTomato^+^FOXF1: GFP ESCs. TdTomato^+^, CD1 males are crossed with FOXF1: GFP, C57BL/6 females and ESC clones are isolated from the blastocyst stage. Clones are screened and expanded for downstream experiments. **(B)** Brightfield and immunofluorescent images show the morphology of colonies and constitutively expressing tdTomato in the selected tdTomato^+^FOXF1: GFP ESC line (line 4). Scale bars = 50 μm. **(C)** Schematic diagram shows the directed differentiation protocol used to produce c-KIT^+^FOXF1^+^ endothelial progenitor cells from line 4 ESCs. Additionally, the schematic shows the workflow of BPD injury and EPC injection where pups are exposed to 75% O_2_ for 7 days from P1-P7. Pups are injected retro orbitally with bulk 60,000 differentiated ESCs per mouse and left to recover in room air until harvest at P15. *N* = 4 mice per group. **(D)** Dot plots show the presence tdTomato^+^ cells in the pulmonary endothelial (CD31^+^CD45^−^) compartment of mice post-EPC injection, compared to control non-injected mice. Measurement of mean fluorescent intensity (MFI) confirms tdTomato expression within this pulmonary endothelial population. *N* = 4 mice per group. **(E)** Immunostaining shows single-cell integration of tdTomato^+^ cells in the distal vascular network of EPC-injected mice with counterstain DAPI and co-expression of tdTomato with endothelial marker Endomucin. Scale bars = 50 μm (top), 20 μm (bottom) Representative images shown, *N* = 4. Illustrations created with BioRender.com.

To evaluate the *in vivo* engraftment potential of ESC-derived cells after directed differentiation into EPCs, we utilized a murine tdTomato^+^FOXF1:GFP ESC line (Line 4) (Figure 2A), which was described and characterized previously (Wang et al., 2021). TdTomato is constitutively expressed which allows us to lineage-trace ESC-derived donor cells *in vivo*, in case the cells differentiate and lose the FOXF1:GFP reporter. Line 4 ESCs exhibited appropriate growth *in vitro*, with well-defined colonies as visualized by brightfield microscopy and tdTomato fluorescence (Figure 2B). Line 4 ESCs were subject to differentiation while newborn murine pups were placed in 75% hyperoxia chambers for 7 days (postnatal days P1-P7) to induce a BPD-like lung injury (Figure 2C). On P8, pups were removed from the chamber and 60,000 differentiated cells were injected retro orbitally. Pups were then left in room air (RA) conditions and allowed to recover for 7 days (P15). On P15, mice were harvested and tdTomato^+^ donor cells could be observed visually with the naked eye, prior to lung tissue processing (Supplementary Figure S4). Flow cytometry analysis revealed that nearly 4% of all CD31^+^CD45^−^ endothelial cells in the murine lung were tdTomato^+^ (Figure 2D). TdTomato was undetectable in non-injected littermates (controls) (Figure 2D). Thus, engrafted tdTomato^+^ cells originated from donor ESCs. Immunofluorescent staining of lung frozen tissues showed two types of donor engraftment. The first, is the expected single-cell engraftment, where elongated tdTomato^+^ cells were seen incorporated into the alveolar microvasculature and co-expressing the endothelial marker endomucin (Figure 2E), consistent with the endothelial origin of these cells. This type of engraftment was similar to the engraftment of endogenous lung c-KIT^+^FOXF1^+^ EPCs into neonatal lung tissue (Ren et al., 2019). The second type of engraftment consisted of clusters of tdTomato^+^ cells in the distal region of the lung. Immunofluorescent staining revealed co-expression of tdTomato with early lung epithelial markers such as NKX2.1 (TTF1), SRY-box transcription factor 2 (SOX2), and SRY-box transcription factor 9 (SOX9). The expression pattern of these markers also resembled that of an early developing lung, with SOX2 marking proximal-like tubule structures (Supplementary Figures S4B, S5B) and SOX9 marking distal tissue surrounding SOX2-positive tubules (Supplementary Figure S4B). TdTomato^+^ cells were restricted to lung tissue and were not found in the heart, liver, kidney, (Supplementary Figure S4C), or bone marrow (Supplementary Figure S4D). To examine the structure of these cell clusters in closer detail, we performed H&E staining and found that clusters could be easily identified in EPC-injected lungs but not in lungs of controls (Supplementary Figure S5A). Interestingly, unlike solid tumors, these structures were not solid masses but instead exhibited areas of uncompacted cells which resembled distal embryonic lung tissue (Supplementary Figure S5A). Further immunofluorescent characterization identified expression of Ki-67 and Caspase-3 (Supplementary Figures S6A, B) identifying the presence of proliferative and apoptotic cells. Co-expression of tdTomato with endothelial marker endomucin and smooth muscle marker alpha-smooth muscle actin (α-SMA), demonstrated that endothelial and smooth muscle cells in these clusters were derived from donor ESCs (Supplementary Figures S6C, D). Thus, bulk injection of differentiated ESCs, results in both single-cell and multicell engraftment which is restricted to the pulmonary tissue and expresses early lung development markers. These results suggest that donor cells contain both endothelial progenitors which give rise to mature endothelial cells capable of single-cell engraftment in the distal lung as well as early lung progenitors with the capacity to generate multiple lung cell types in the recipient lung.

### Addition of BMP9 to differentiation media results in efficient and stable generation of c-KIT^+^FOXF1^+^ EPCs

**FIGURE 3:**
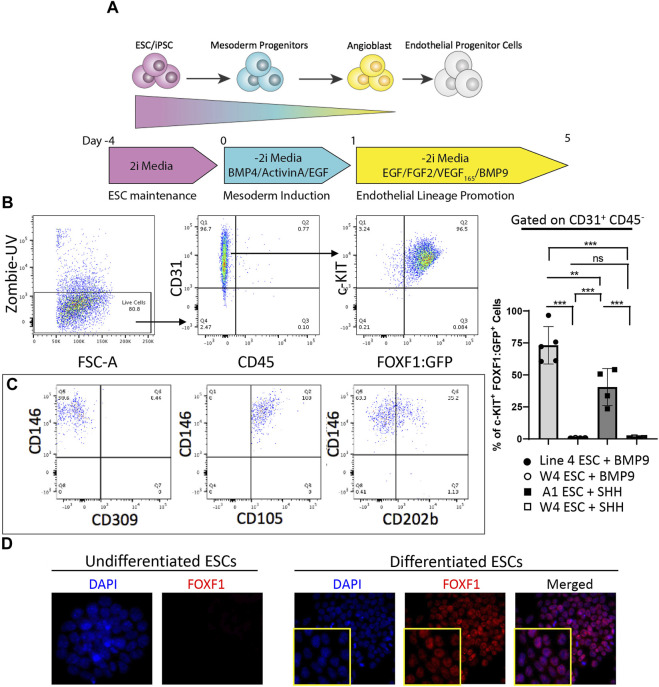
Addition of BMP9 to differentiation media results in efficient and stable generation of c-KIT^+^FOXF1^+^ EPCs. **(A)** The schematic shows the directed differentiation protocol starting with line 4 ESC maintenance, mesoderm induction, and endothelial lineage promotion with the modification of added Bone morphogenic protein 9 (BMP9) to promote efficient and stable expression of FOXF1. **(B)** Flow cytometry analysis shows the result of line 4 ESC differentiation. Differentiated line 4 ESCs produce mainly endothelial cells as marked by CD31^+^CD45^−^ cell surface expression. Within the endothelial cell compartment, a greater percentage of cells become c-KIT^+^FOXF1^+^ EPCs after addition of BMP9. This experiment was repeated 5 time. **(C)** Further FACS analysis shows that in vitro-generated c-KIT^+^FOXF1^+^ EPCs exhibit appropriate endothelial marker expression such as Cluster of differentiation 146 (CD146), Endoglin (ENG, CD105), and Angiopoietin-1 receptor (CD202b). **(D)** Immunostaining reveals that undifferentiated ESCs lack expression of FOXF1 as compared to nuclear expression found in differentiated and FACS-sorted EPCs. Image magnification is × 10. This experiment was repeated 4 times with similar results. Values are shown as mean ± SD. **p < 0.01, ***p < 0.001, ns is not significant.

Previous work demonstrated that c-KIT^+^FOXF1^+^ EPCs can be activated via BMP9/ACVRL1/SMAD1 signaling (Wang et al., 2022). Therefore, we used this published information to increase the generation of c-KIT^+^FOXF1^+^ EPCs while reducing the generation of early pulmonary progenitors. BMP9 was added to the culture media, whereas SHH was removed from the media to prevent generation of nonmature mesenchyme and smooth muscle cells from ESCs (Figure 3A). ESC differentiation and subsequent flow cytometry analysis showed that this protocol modification successfully produced CD31^+^CD45^−^ cells, specifically a higher percentage of c-KIT^+^FOXF1^+^ EPCs as compared to the previous protocol (Figure 3B). Analysis of cell surface markers by FACS revealed that in vitro-generated c-KIT^+^FOXF1^+^ EPCs exhibit appropriate endothelial marker expression such as Cluster of differentiation 146 (CD146), Endoglin (ENG, CD105), and Angiopoietin-1 receptor (CD202b) (Figure 3C) which was similar to the expression pattern of these marker proteins in endogenous lung c-KIT^+^FOXF1^+^ EPCs (Wang et al., 2022). Immunofluorescent staining of cells grown in chamber slides, confirmed the presence of endogenous FOXF1 protein in differentiated cells, compared to no expression in their undifferentiated ESC counterparts (Figure 3D). Therefore, exclusion of SHH and addition of BMP9 to the protocol results in a more robust generation of c-KIT^+^FOXF1^+^ EPCs with appropriate expression of cell surface markers, including CD31, c-KIT, CD146, CD105, and CD202b.

### Purified donor c-KIT^+^FOXF1^+^ EPCs are capable of single-cell integration in the alveolar region of BPD mice

**FIGURE 4:**
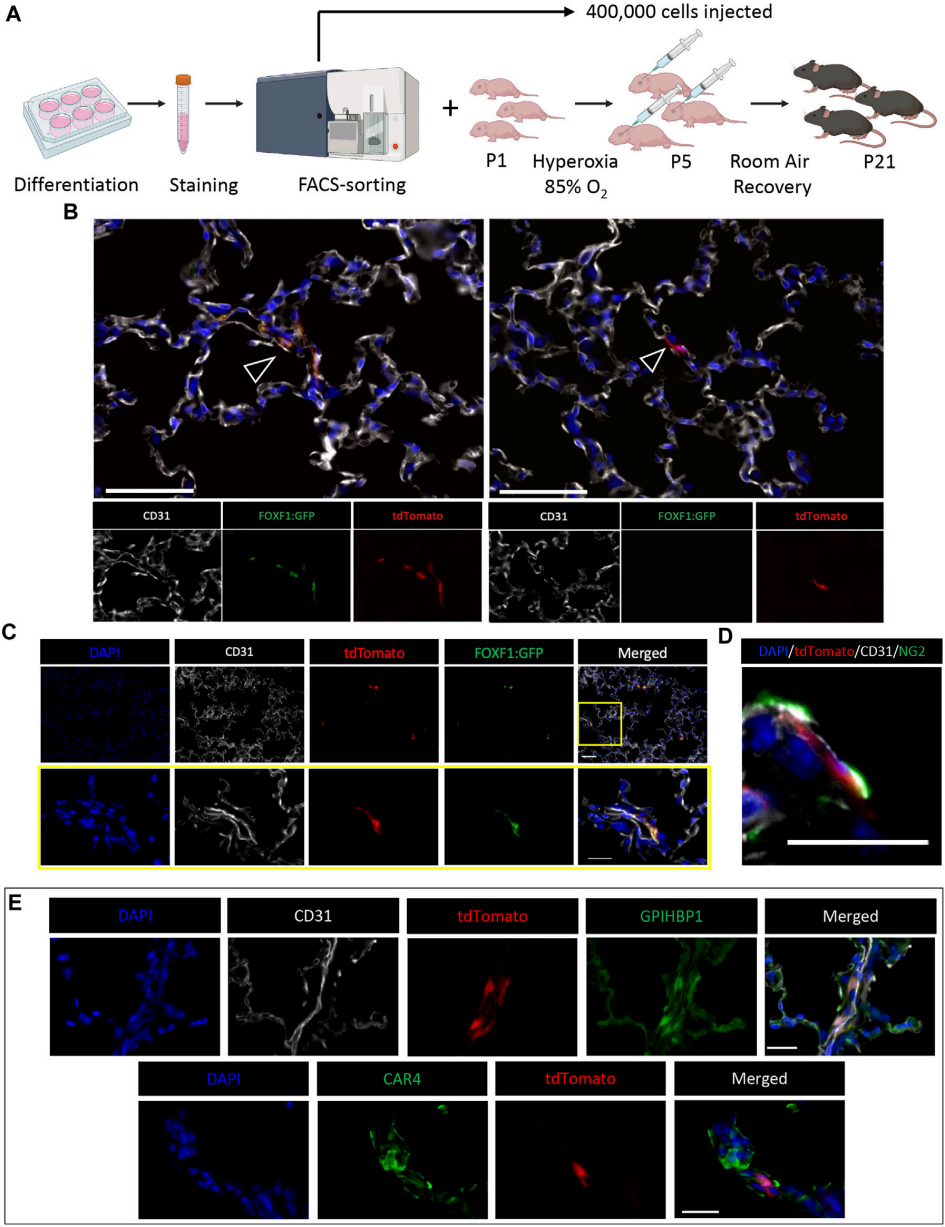
ESC-derived c-KIT^+^FOXF1^+^ EPCs are capable of single-cell integration in the alveolar region of BPD mice. **(A)** Schematic shows the workflow of EPC isolation, BPD injury, cell injection, and mouse recovery. Line 4 ESCs are differentiated according to the protocol, harvested, stained, and FACS-sorted for injection. Simultaneously, P1 pups are placed in 85% O_2_ hyperoxia chambers to induce a BPD-like lung injury. After 5 days (P5) pups are removed from hyperoxia and injected retro orbitally with 400,000 sorted c-KIT^+^FOXF1^+^ EPCs per mouse and left to recover in room air conditions until harvest at P21. *N* = 4 mice per group. **(B)** Immunostaining shows single cell integration of tdTomato^+^ donor cells in alveoli. Regions of integration contain both tdTomato^+^GFP^+^ and tdTomato^+^GFP^−^ cells (arrowheads). Scale bars = 50 μm. **(C)** Co-staining with endothelial marker CD31, shows that tdTomato^+^GFP^+^ engrafted cells express CD31. Scale bars = 50 μm (top), 20 μm (bottom). **(D)** TdTomato^+^GFP^+^ cells express endothelial marker CD31 and are in close spatial association with pericytes, marked by Neuron-glial antigen 2 (NG2) expression. Scale bar = 20 μm. **(E)** Immunostaining shows that injected EPCs can differentiate into both CAP1 and CAP2 cells in the alveolar region, as identified by co-expression of tdTomato with glycosylphosphatidylinositol anchored high density lipoprotein binding protein 1 (GPIHBP1) and Carbonic anhydrase 4 (CAR4). Scale bars = 20 μm. Representative images shown, *N* = 4. Illustrations created with BioRender.com.

To induce neonatal lung injury and promote single cell engraftment by evading the developing immune system in neonatal mice, we chose to modify the hyperoxia regimen such that we could obtain the same level of lung injury but inject mice at an earlier developmental timepoint (P5 instead of P7). Therefore, we placed newborn pups (P1) in 85% hyperoxia chambers for 5 days (P5) (Figure 4A). Simultaneously, ESCs were differentiated into c-KIT^+^FOXF1^+^ EPCs *in vitro* (Supplementary Figure S7A). Using FACS-sorting for tdTomato and the FOXF1:GFP transgene, we purified c-KIT^+^FOXF1^+^ EPCs and injected them retro orbitally into hyperoxia-injured P5 pups. We also increased the injected cell quantity to 400,000 cells per mouse (Supplementary Figure S7B). To improve engraftment, mice were allowed extended time to recover in room air conditions and were harvested 3 weeks after birth (P21). Immunofluorescent staining of c-KIT^+^FOXF1^+^ EPC-injected mice revealed the integration of ESC-derived donor cells in the alveolar region of recipient mice (Supplementary Figures S7C, D). Furthermore, regions of identified integration contained both tdTomato^+^ GFP^+^ and tdTomato^+^ GFP^−^ cells, indicating that some of the donor EPCs have lost FOXF1 expression and undergone differentiation upon incorporation into the alveolar microvasculature (Figure 4B). Donor cells, marked with tdTomato and GFP, colocalized with expression of endothelial marker CD31 (Figure 4C), confirming the endothelial cell lineage identity. Donor endothelial cells were found in close association with pericytes, marked by Neuron-glial antigen 2 (NG2) (Figure 4D), a specific pericyte marker (Ozerdem et al., 2002; Fukushi et al., 2004). Thus, donor EPCs engrafted into the alveolar microvasculature, as demonstrated both by CD31 expression and spatial association with pericytes.

Recently, Gillich et al. showed that the alveolar capillary endothelium is heterogenous and comprised of two main specialized cell types, general capillary cells (gCaps or CAP1) and aerocytes (aCaps or CAP2) (Gillich et al., 2020). CAP1 cells express markers such as APLNR and GPIHBP1, whereas CAP2 cells express APLN and CAR4 (Gillich et al., 2020). ESC-derived donor c-KIT^+^FOXF1^+^ EPCs had the capacity to differentiate into both CAP1 and CAP2 populations in neonatal lungs injured by hyperoxia, as demonstrated by immunostaining and colocalization of tdTomato with GPIHBP1 and CAR4 (Figure 4E). Thus, injection of FACS-sorted c-KIT^+^FOXF1^+^ EPCs results in stable donor cell integration, wherein engrafted cells integrate appropriately into the alveolar microvasculature and express endothelial-specific markers including those of both CAP1 and CAP2 populations.

### Cell therapy with ESC-Derived c-KIT^+^ FOXF1^+^ EPCs increase capillary density and alveolarization after neonatal hyperoxic injury

**FIGURE 5:**
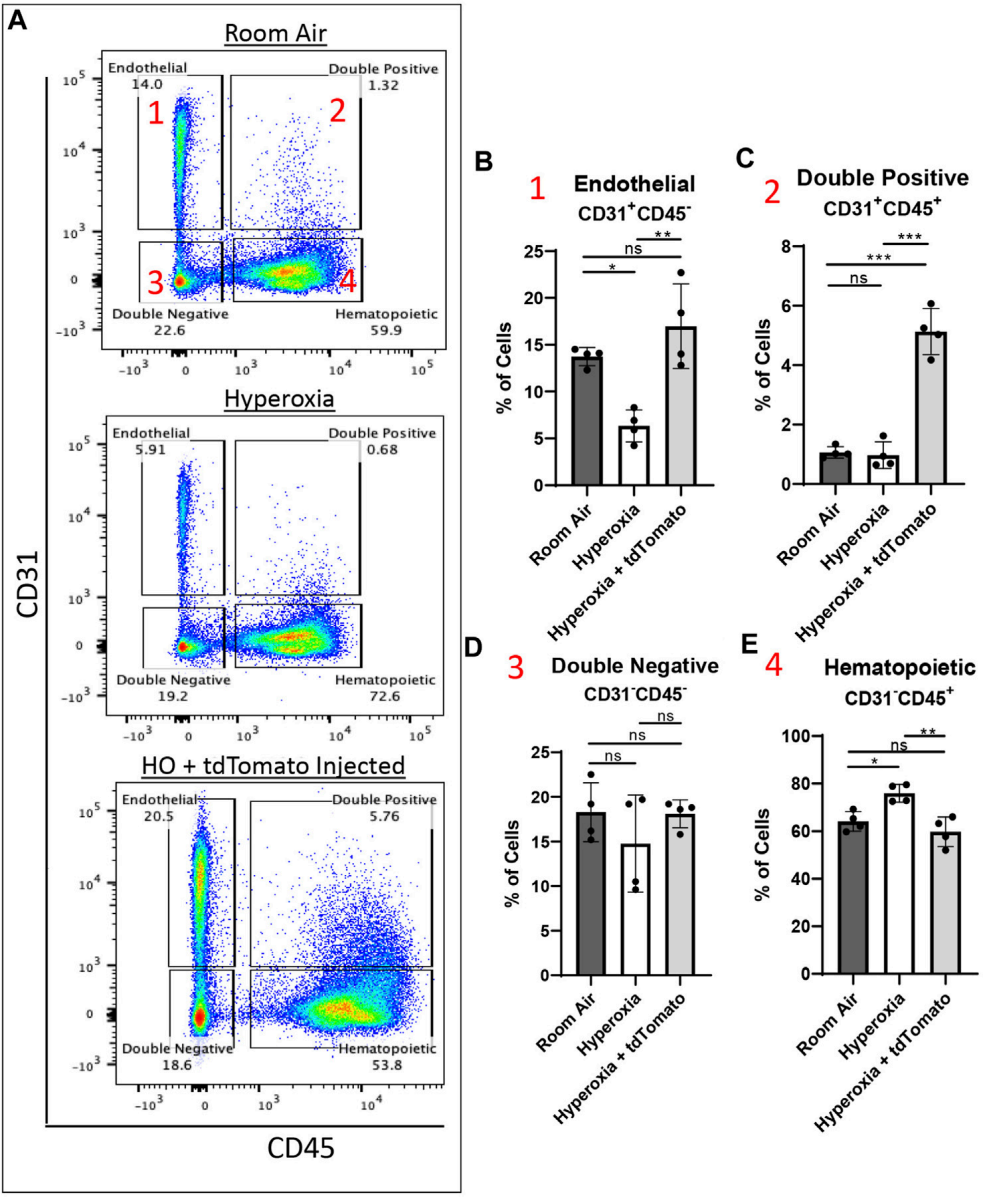
Cell therapy with ESC-derived c-KIT^+^FOXF1^+^ EPCs increases the number of endothelial cells, circulating EPCs, and decreases inflammatory cells in lungs of hyperoxia-injured mice. **(A)** Dot plots show the gating strategy used to subdivide the major cell types in the murine lung across room air, hyperoxia (HO), and hyperoxia with EPC-injected groups. The cells can be subdivided into four major groups: 1 Endothelial (CD31^+^CD45^−^), 2 Double positive (CD31^+^CD45^+^), 3 Double negative (CD31^−^CD45^−^), 4 Hematopoietic (CD31^−^CD45^+^). The cell percentage was quantified for **(B)** Endothelial, **(C)** Double positive, **(D)** Double negative, and **(E)** Hematopoietic cell compartments using *N* = 4 mice per group. Values are shown as mean ± SD *p < 0.05, **p < 0.01, ***p < 0.001, ns is not significant.

To evaluate the effects of c-KIT^+^FOXF1^+^ EPC engraftment on lung repair after neonatal hyperoxic injury, we used flow cytometry and compared cell populations in the lungs of mice exposed to either room air (RA), hyperoxia, or hyperoxia-injured mice receiving the EPC therapy (Supplementary Figure S8A). Using CD31 and CD45 cell surface markers, major cell types in the lung were subdivided into 4 main groups: Endothelial (CD31^+^CD45^−^), Hematopoietic (CD31^−^CD45^+^), Double positive cells containing circulating EPCs (CD31^+^CD45^+^), and Double negative cells containing all other cell types (CD31^−^CD45^−^) (Figure 5A). When comparing the cell compartments, we found that as expected, hyperoxic injury significantly decreased the percentage of cells in all compartments except hematopoietic which saw a significant increase in cell percentage compared to RA (Figure 5E), consistent with increased lung inflammation after neonatal hyperoxic injury (Xia et al., 2015). After EPC therapy, hyperoxia-injured mice experienced significant increases in all cell compartments except the hematopoietic which returned to baseline RA levels (Figures 5B–E), suggesting a decrease in the inflammatory response post-injury. Further studies are needed to confirm a decrease in the inflammatory response of these mice. Importantly, hyperoxia-injured mice treated with EPCs had a significant increase in the percentage of endothelial cells, restoring the endothelial compartment to levels seen in the RA control group (Figure 5B). Interestingly, the hyperoxia-injured mice treated with EPCs showed a significant increase in the double positive (CD31^+^CD45^+^) cell population, significantly greater than that of both hyperoxia-injured and RA control groups (Figure 5C). Several groups have identified and classified circulating endothelial progenitor cells (circulating EPCs) as having both characteristics of endothelial and hematopoietic cells and expressing both CD31 and CD45 (Rohde et al., 2006; Rohde et al., 2007; Asahara et al., 2011; Kolesnichenko et al., 2021). Thus, cell therapy with ESC-derived c-KIT^+^FOXF1^+^ EPCs increases the numbers of endothelial cells and circulating EPCs and decreases the number of inflammatory cells in the lung tissue of hyperoxia-injured mice.

**FIGURE 6:**
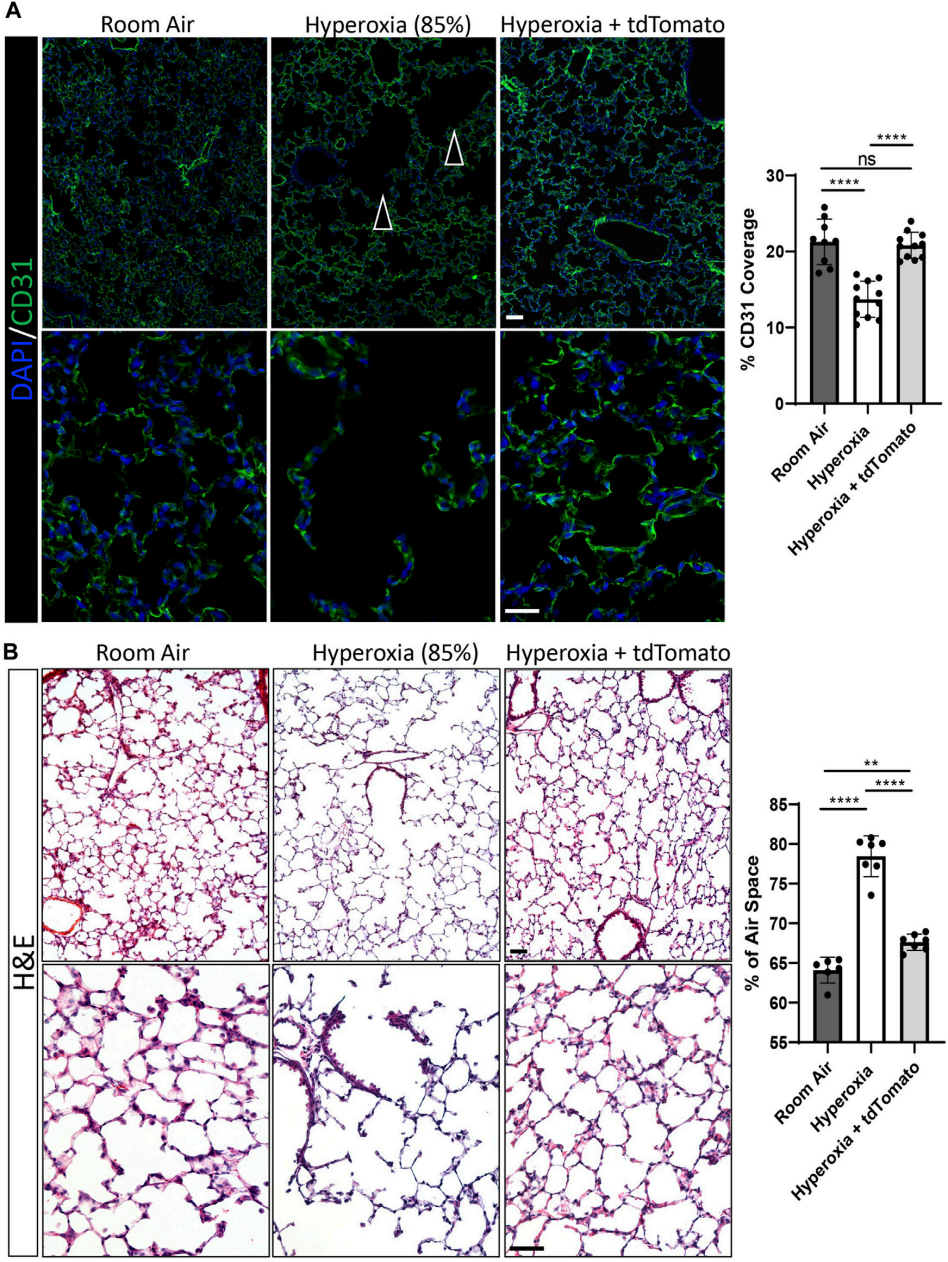
Cell therapy with ESC-derived c-KIT^+^FOXF1^+^ EPCs increases capillary density and improves alveolarization after neonatal hyperoxic injury. **(A)** Immunostaining of frozen lung sections for CD31 show that exposure to 85% O_2_ (hyperoxia) results in a disturbed vascular network (indicated by arrowheads) with reduced endothelial cell coverage. Treatment with ESC-derived c-KIT^+^FOXF1^+^ EPCs restores the vascular network and endothelial cell coverage in alveolar regions to the levels seen in room air controls. **(B)** H&E staining of frozen lung sections show that hyperoxia exposure results in large alveoli and increased air space in the lung. Treatment with ESC-derived c-KIT^+^FOXF1^+^ EPCs decreases average alveolar size to levels seen in room air controls. Five random fields were captured per sample to quantify CD31 coverage and % air space. Values are shown as mean ± SD. *N* = 6–11 mice per group, *p < 0.05, **p < 0.01, ***p < 0.001, ns is not significant. Scale bars = 50 μm.

In addition to quantitative comparisons of respiratory cell populations, we examined if the findings obtained by flow cytometry could be supported by structural examination of lung sections from the same groups. In agreement with the flow cytometry findings, immunofluorescent staining for CD31 showed that hyperoxia-injured mice had significantly less endothelial coverage in alveoli compared to RA controls (Figure 6A). The endothelial coverage was restored to RA levels in hyperoxia-injured mice treated with ESC-derived c-KIT^+^FOXF1^+^ EPCs (Figure 6A). To examine alveolarization, we evaluated the percent of airspace in alveolar regions of all groups and found that hyperoxia-injured mice developed alveolar simplification marked by increased alveolar spaces (Figure 6B). Treatment with ESC-derived c-KIT^+^FOXF1^+^ EPCs significantly decreased the size of the alveolar spaces in hyperoxia-injured mice, reducing the alveolar simplification caused by hyperoxic injury (Figure 6B). Future work should include the use of the flexiVent lung function system to evaluate other clinical parameters of lung function and output. Altogether, treatment with ESC-derived c-KIT^+^FOXF1^+^ EPCs resulted not only in donor cell engraftment into the lung tissue but also in an increase in the total number of endothelial cells and circulating EPCs and a decrease in the number of hematopoietic cells in the hyperoxia-injured lung. Additionally, the c-KIT^+^FOXF1^+^ EPC treatment restored endothelial density and decreased alveolar simplification in the mouse model of BPD.

## Discussion

The survival of extremely premature infants, especially those born before 28 weeks of gestation, continues to increase due to our advances in neonatal medicine. Unfortunately, these infants are at high risk of developing complications with implications that extend into adolescence and adulthood (Eber and Zach, 2001). BPD is the most frequently diagnosed adverse outcome of prematurity (Jensen and Schmidt, 2014). Advances in research and clinical care have led to the introduction of antenatal steroids, surfactant therapy, and novel ventilation regimens. As a result, the presentation of BPD has evolved over time from a mainly fibrotic disease (old BPD) to a disease marked by an arrest in alveolarization (termed as alveolar simplification), respiratory insufficiency requiring mechanical ventilation, and decreased capillary density, in the most severe cases of BPD (new BPD) (Collins et al., 2017). As these premature infants age and as new fragile patient populations emerge, it is integral to explore new scientific avenues in the search for an efficient and effective therapy for patients with chronic neonatal pulmonary diseases such as BPD. In the present study, we use a hyperoxia-induced mouse model of BPD and provide a proof-of-principle that cell therapy with c-KIT^+^FOXF1^+^ EPCs (generated from pluripotent embryonic stem cells *in vitro*) can improve neonatal angiogenesis and alveolarization in the mouse BPD model.

Since their first description in 1997, great strides have been made in the identification and evaluation of new EPC populations and their role in development, disease, and injury repair (Nagaya et al., 2003; He et al., 2004; Takahashi et al., 2004; Zhao et al., 2005; Keighron et al., 2018; Qin et al., 2018; Ren et al., 2019; Wang et al., 2021). One such population is c-KIT^+^FOXF1^+^ tissue-resident EPCs which represent a subset of CAP1 cells residing in the pulmonary microvasculature. Single-cell RNA sequencing data of mouse and human newborn lungs revealed a highly conserved gene signature in c-KIT^+^FOXF1^+^ EPCs which are also highly enriched in expression of FOXF1 downstream target genes (Ren et al., 2019). FOXF1 is an evolutionarily conserved transcription factor which plays an important role in cell proliferation, angiogenesis, lung repair, and regeneration. Point mutations in *FOXF1* such as S52F, result in a severe pediatric lung disease called Alveolar capillary dysplasia with misalignment of pulmonary veins (ACDMPV) which is fatal without neonatal lung transplantation (Towe et al., 2018). Not only is the c-KIT^+^FOXF1^+^ EPC population decreased as a result of haploinsufficiency or endothelial-specific deletion of *Foxf1* but this EPC population is also highly sensitive to hyperoxia exposure as seen in both human and mouse BPD lungs (Balasubramaniam and Ingram, 2009; Ren et al., 2019).

Herein, we take a cellular therapy approach to lung regeneration in a hyperoxia-induced mouse model of BPD using *in vitro* generated c-KIT^+^FOXF1^+^ EPCs. First, using a novel, genetically modified ESC line with GFP knocked in to the *Foxf1* locus we employed a modified directed differentiation protocol and were able to successfully generate CD31^+^CD45^−^ endothelial cells specifically, a c-KIT^+^FOXF1^+^ EPC cell population. Bulk injection of in vitro-generated EPCs with a tdTomato tracer, into hyperoxia-injured neonatal mice resulted in nearly 4% tdTomato^+^ cells in the endothelial compartment of recipient mice. Interestingly, this engraftment was both single- and multicellular. TdTomato^+^ cell clusters were only found in lung tissue and expressed markers relevant to early lung development such as TTF1, SOX9, and SOX2. H&E staining revealed more similarities to early lung tissue than those of solid tumors, with regions resembling both uncompacted distal lung and proximal lung tubules. Therefore, bulk injection of in vitro-derived EPCs likely contained c-KIT^+^FOXF1^+^ EPCs which gave rise to endothelial cells as well as other early lung progenitors with the capacity to differentiate into multiple lung-specific cell types, including epithelial cells expressing TTF1, SOX9, and SOX2.

Addition of BMP9 and FACS-sorting of cells after differentiation, resulted in a homogenous and stable population of c-KIT^+^FOXF1^+^ EPCs. These cells were capable of single cell engraftment in the pulmonary microvasculature of recipient mice, a finding consistent with engraftment of lung-derived (endogenous) c-KIT^+^FOXF1^+^ EPCs (Ren et al., 2019). Injected cells expressed lung endothelial markers such as CD31, FOXF1:GFP, GPIHBP1, CAR4, and also associated closely with pulmonary pericytes, demonstrating proper engraftment by both gene expression and association with endogenous cell types. Importantly, treatment with c-KIT^+^FOXF1^+^ EPCs increased the number of endothelial cells in hyperoxia-injured lungs, restoring levels to those of RA control animals. The EPC treatment also decreased the hematopoietic response to hyperoxia injury and increased the number of CD31^+^CD45^+^ cells in the recipient’s lungs which likely represent a population of circulating EPCs (Kolesnichenko et al., 2021) that further aide in lung repair and regeneration. Further investigation is needed in this area to determine the exact identity and contribution of CD31^+^CD45^+^ cells to therapeutic effects of EPC therapy on lung regeneration in BPD. Consistent with the effects of endogenous c-KIT^+^FOXF1^+^ EPCs (Ren et al., 2019), treatment with ESC-derived c-KIT^+^FOXF1^+^ EPCs (via *in vitro* cell differentiation) was successful in increasing endothelial coverage, restoring the density of the pulmonary microvasculature, and decreasing the size of alveolar spaces in the mouse BPD model.

Several limitations are present in the current study. The first is the animal model of BPD which although well-established does not perfectly mimic a patient’s presentation with the disease. BPD is multifactorial in nature and exposure to hyperoxia alone fails to take into account complications such as infection, preeclampsia, and genetic predispositions. Second, this work was performed using mouse ESC lines, and mouse recipients. To effectively bridge the gap between bench and bedside, further work is needed to move this concept to the use of induced pluripotent stem cells (iPSCs), where somatic cells can be taken from a patient, reprogrammed to iPSCs and then differentiated to c-KIT^+^FOXF1^+^ EPCs for further testing and characterization. Additionally, donor-recipient matching should be considered and tested in long-term studies using ESCs/iPSCs-derived EPCs. Lastly, future work should focus on an in-depth comparison between in vitro-generated and endogenous (lung-derived) c-KIT^+^FOXF1^+^ EPCs to determine if in vitro-generated cells use the same mechanisms to improve lung regeneration and decrease lung inflammation after neonatal lung injury.

In summary, we have created a novel ESC line and modified existing ESC differentiation protocols to generate functional c-KIT^+^FOXF1^+^ EPCs from pluripotent ESCs *in vitro*. Transplantation of FACS-sorted, ESC-derived EPCs into the systemic circulation of hyperoxia-injured mice resulted in cell engraftment in the pulmonary microvasculature and improved lung angiogenesis and alveolarization after injury. Our studies highlight the potential of ESC/iPSC-derived endothelial progenitor cells to gain insight into the process of lung regeneration in a murine, hyperoxia-induced model of BPD.

## Data Availability

The raw data supporting the conclusion of this article will be made available by the authors, without undue reservation.
