# Supplementary figures and images for: Endothelial progenitor cells derived from embryonic stem cells prevent alveolar simplification in a murine model of bronchopulmonary dysplasia
Source: Front Cell Dev Biol. 2023 Jun 9;11:1209518. doi: 10.3389/fcell.2023.1209518 (PMC10289167; doi:10.3389/fcell.2023.1209518)

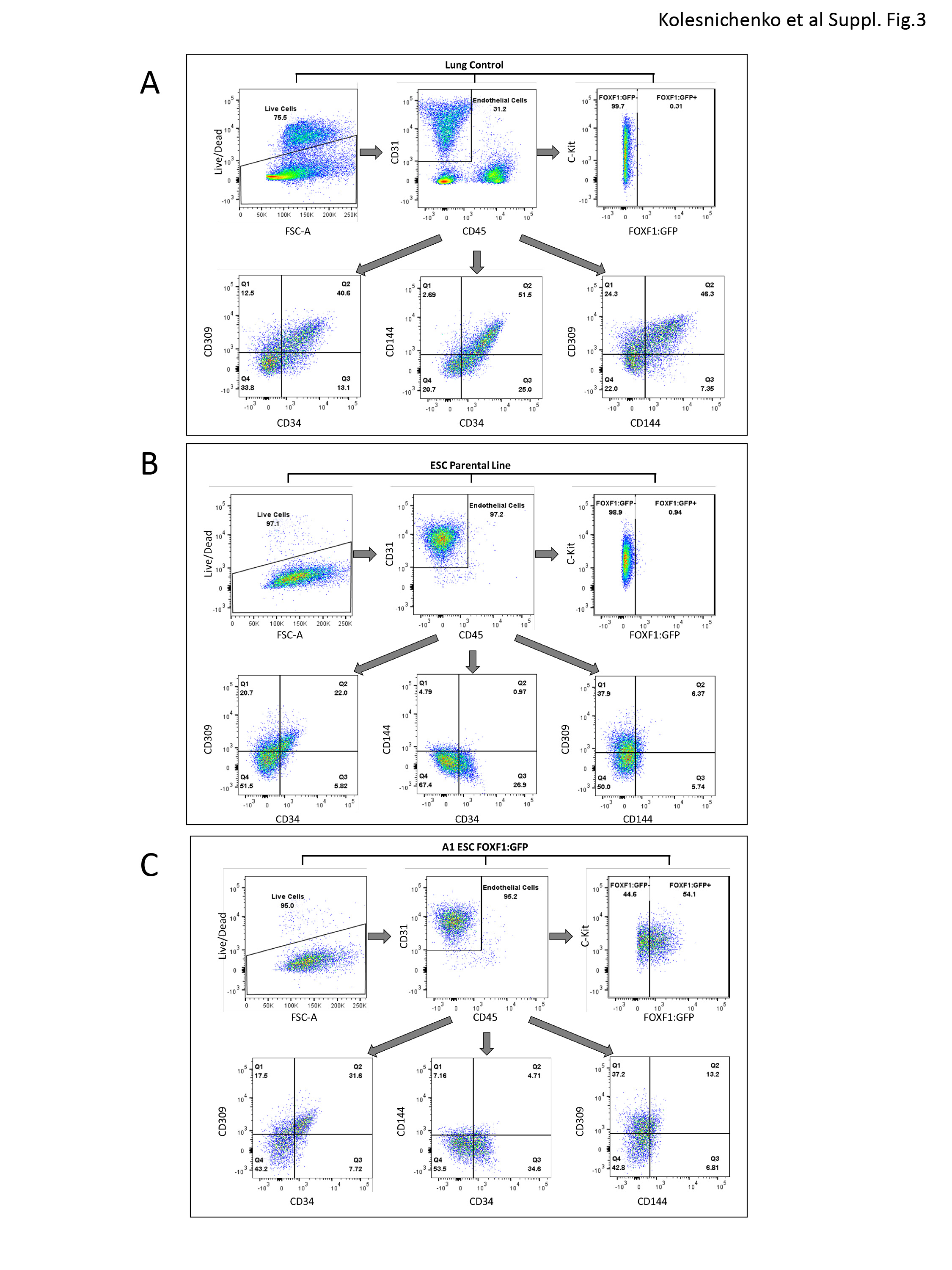

Supplement: Supplementary file 1 [file Image3.JPEG]

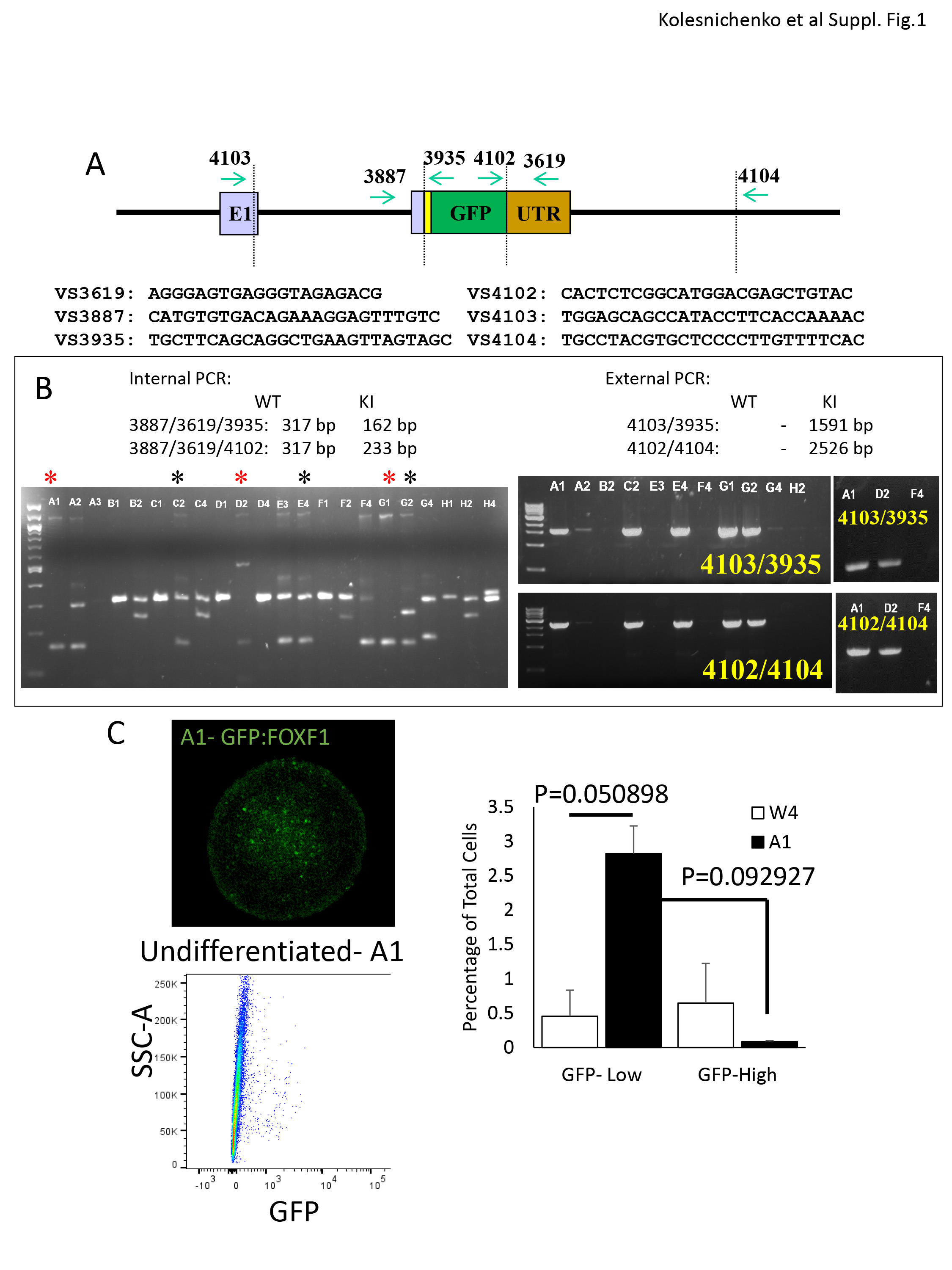

Supplement: Supplementary file 4 [file Image1.JPEG]

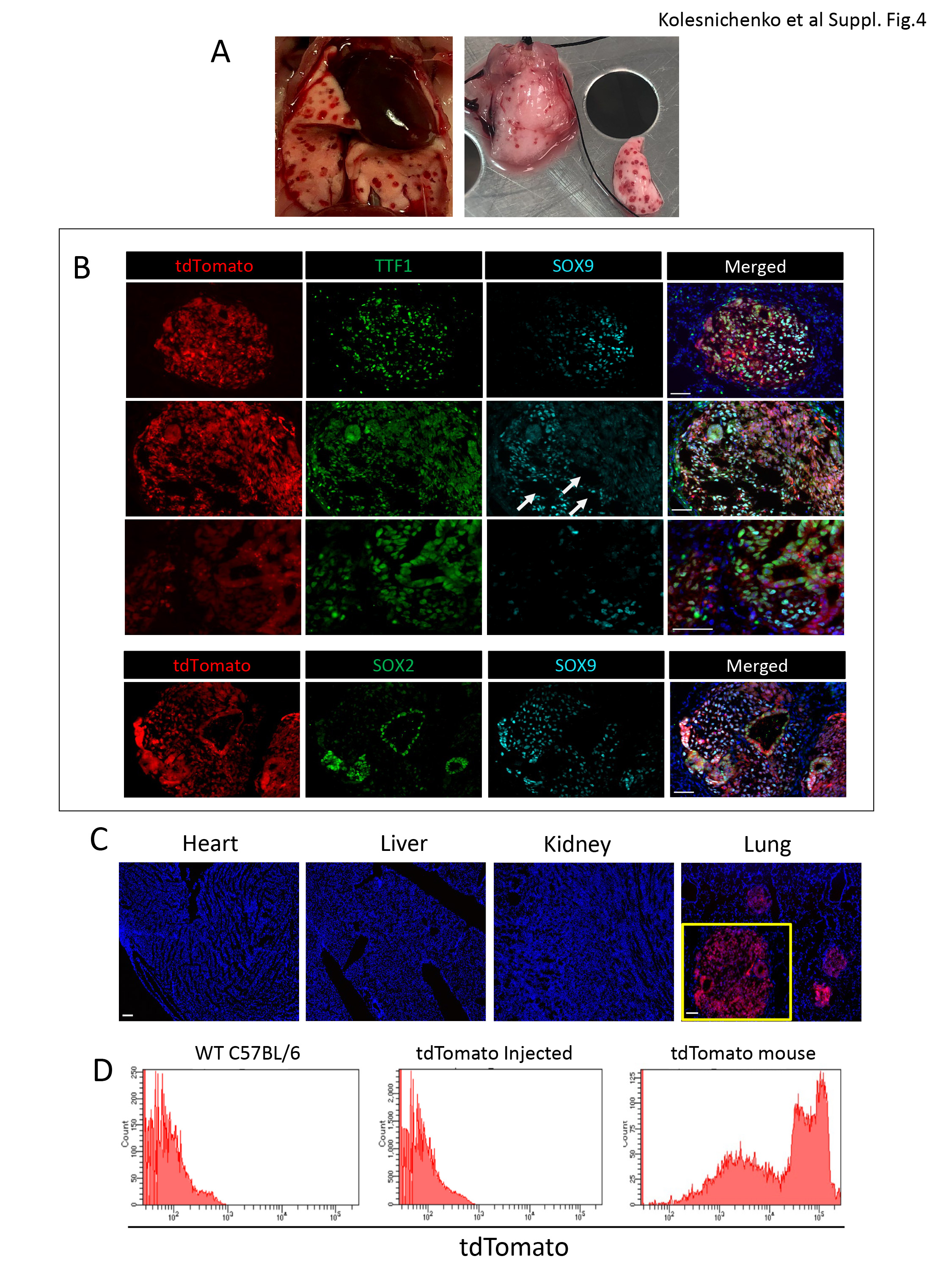

Supplement: Supplementary file 5 [file Image4.JPEG]

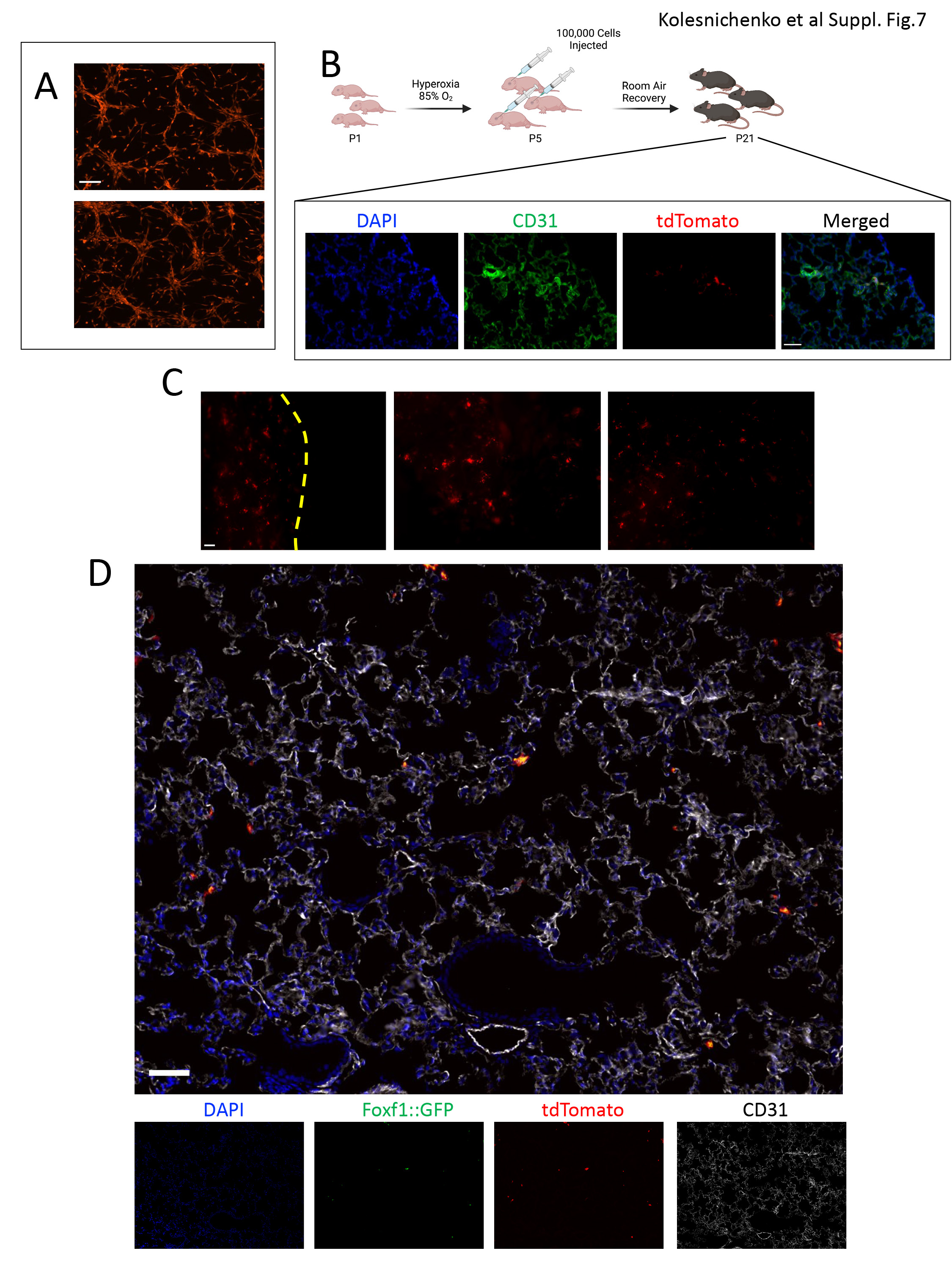

Supplement: Supplementary file 6 [file Image7.JPEG]

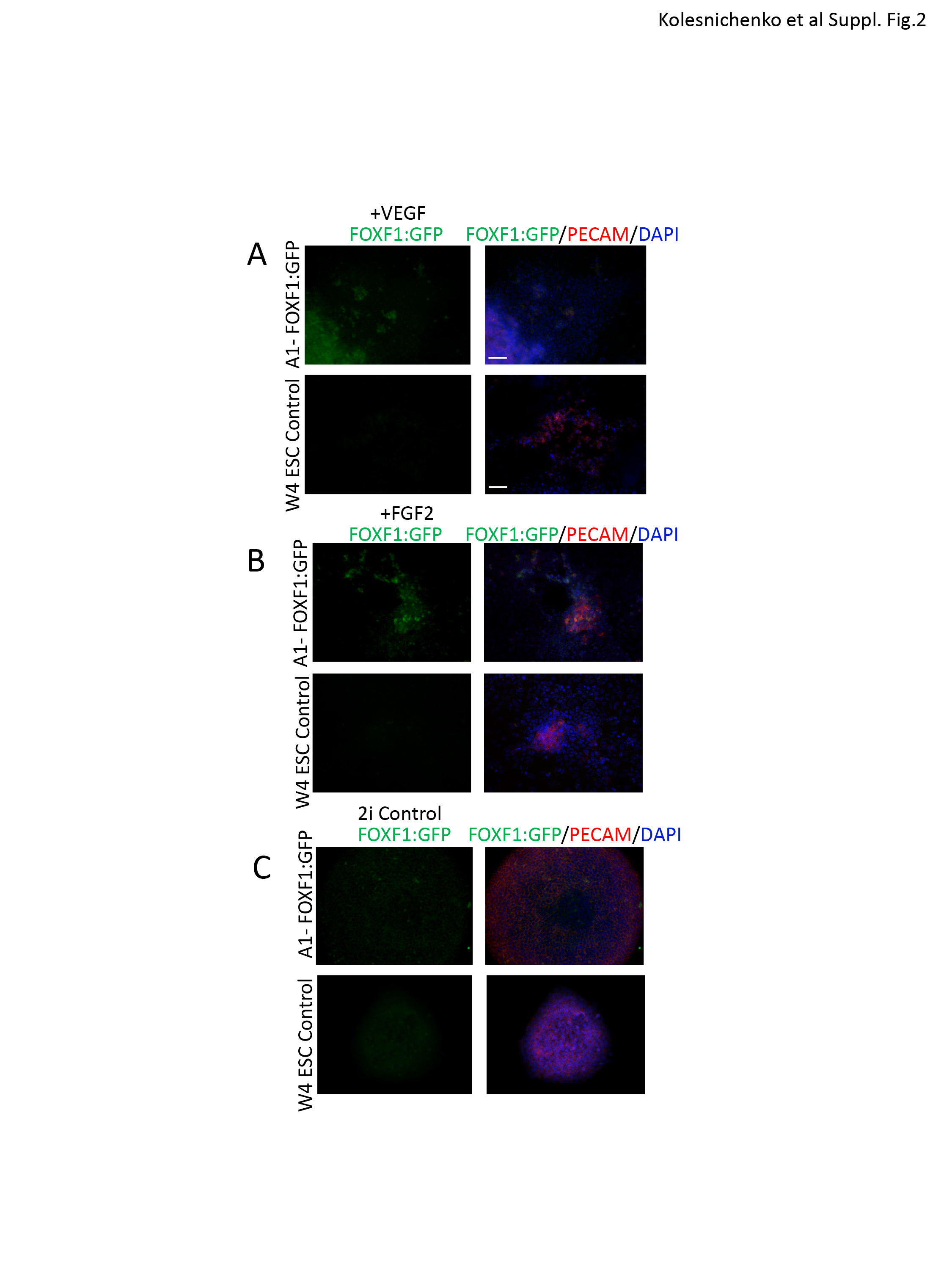

Supplement: Supplementary file 7 [file Image2.JPEG]

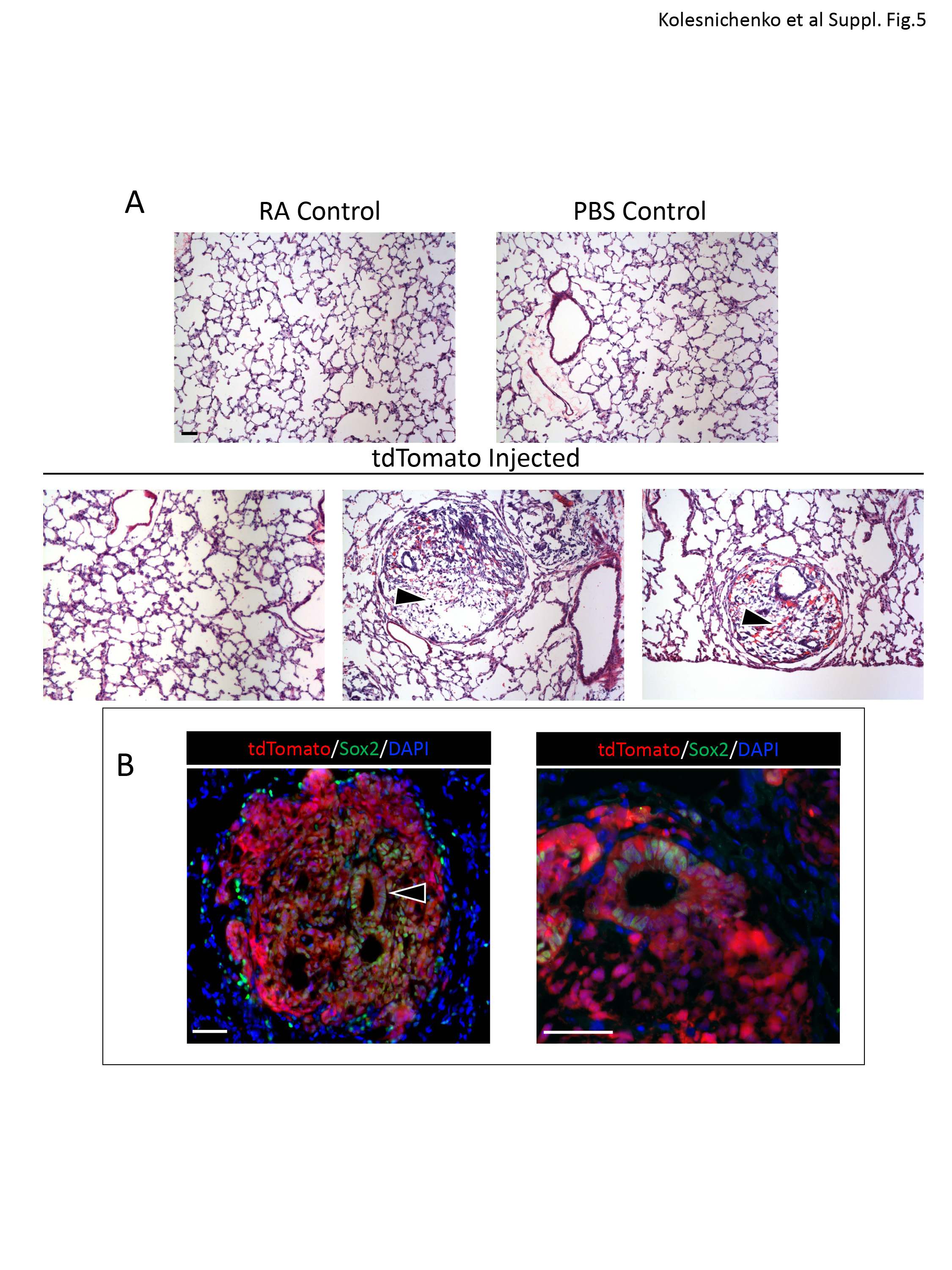

Supplement: Supplementary file 8 [file Image5.JPEG]

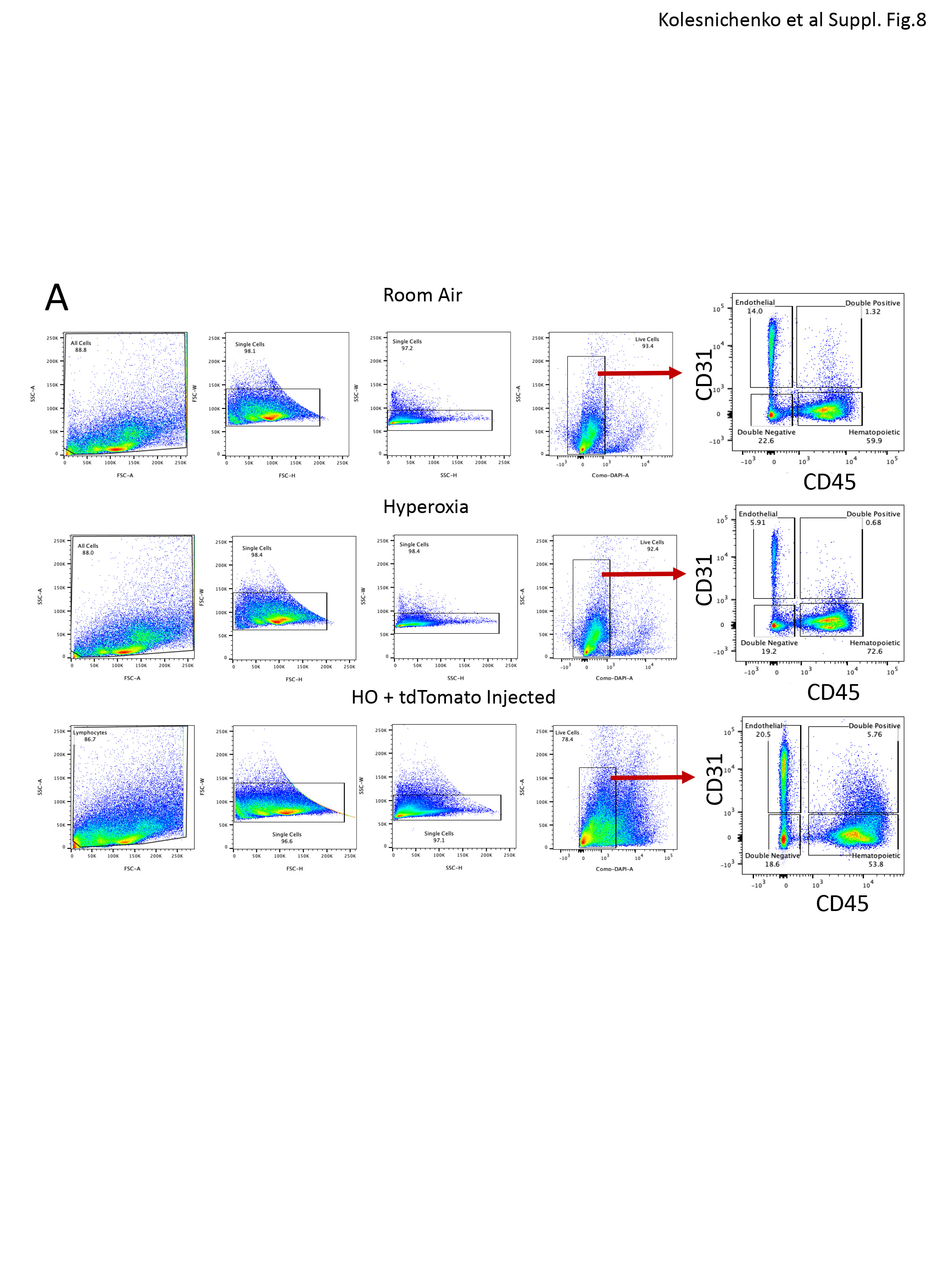

Supplement: Supplementary file 9 [file Image8.JPEG]

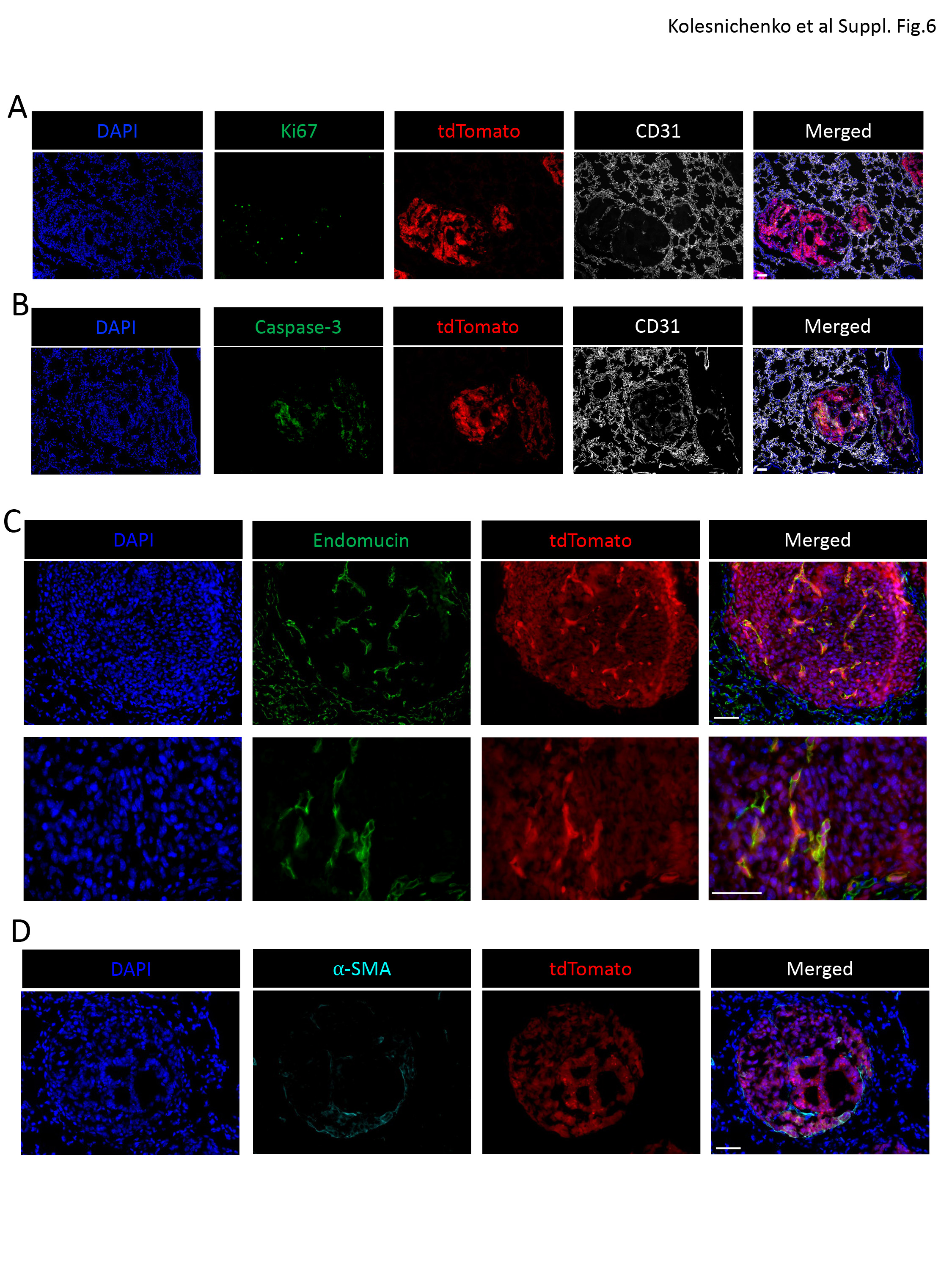

Supplement: Supplementary file 10 [file Image6.JPEG]
