# Supplementary material for: Endothelial progenitor cells derived from embryonic stem cells prevent alveolar simplification in a murine model of bronchopulmonary dysplasia
Source: Front Cell Dev Biol. 2023 Jun 9;11:1209518. doi: 10.3389/fcell.2023.1209518 (PMC10289167; doi:10.3389/fcell.2023.1209518)
Supplement: Supplementary file 2 [file Table1.DOCX]

Supplementary Material

Endothelial Progenitor Cells Derived from Embryonic Stem Cells Prevent Alveolar Simplification Caused by Neonatal Hyperoxic Injury

Olena A. Kolesnichenko, Hannah M. Flood, Yufang Zhang, Hayde K. Cuervo Jimenez, Tanya V. Kalin and Vladimir V. Kalinichenko^*^

*** Correspondence:**

Dr. Vladimir V. Kalinichenko

Phoenix Children’s Health Research Institute

University of Arizona College of Medicine – Phoenix

Phoenix, AZ, United States
Email: vkalin@arizona.edu

**SUPPLEMENTAL FIGURE LEGENDS:**

| Clone | Allele alteration | Sequence |
| --- | --- | --- |
| WT | None | GCATCCCTCGGTATCACTCACAGTCGCCCAGCATGTGTGACAGAAAGGAGTTTGTCTTCTCTTTCAATGCCATGGCCTCTTCTTCTATGCATACAACAGGCGGAGGATCTTACTATCACCAGCAGGTCACCTACCAAGACATCAAGCCGTGTGTGATG**TGA**GGTGAGGCCACGGGGCCCTCCAGCCCAGCCTGGCCGGCCCAGGGACCAGGAGCCCACCGCCACAAACTGCTTTACTCTGGAGGTATAACCCGTCAGCAAGTGAAAAGGGATAGCCCCACCCCTAACGGATTATTTGTAAAGAAAATCCCAACACAGACTGGGAGCAGCGTCTCTACCCTCACTCCCTCA |
| C2 | 1bp insertion | GCATCCCTCGGTATCACTCACAGTCGCCCAGCATGTGTGACAGAAAGGAGTTTGTCTTCTCTTTCAATGCCATGGCCTCTTCTTCTATGCATACAACAGGCGGAGGATCTTACTATCACCAGCAGGTCACCTACCAAGACATCAAGCCGTGTGaTGATG**TGA**GGTGAGGCCACGGGGCCCTCCAGCCCAGCCTGGCCGGCCCAGGGACCAGGAGCCCACCGCCACAAACTGCTTTACTCTGGAGGTATAACCCGTCAGCAAGTGAAAAGGGATAGCCCCACCCCTAACGGATTATTTGTAAAGAAAATCCCAACACAGACTGGGAGCAGCGTCTCTACCCTCACTCCCTCA |
| E4 | 5bp deletion | GCATCCCTCGGTATCACTCACAGTCGCCCAGCATGTGTGACAGAAAGGAGTTTGTCTTCTCTTTCAATGCCATGGCCTCTTCTTCTATGCATACAACAGGCGGAGGATCTTACTATCACCAGCAGGTCACCTACCAAGACATCAAG~~C.CGTGT~~.GTGATG**TGA**GGTGAGGCCACGGGGCCCTCCAGCCCAGCCTGGCCGGCCCAGGGACCAGGAGCCCACCGCCACAAACTGCTTTACTCTGGAGGTATAACCCGTCAGCAAGTGAAAAGGGATAGCCCCACCCCTAACGGATTATTTGTAAAGAAAATCCCAACACAGACTGGGAGCAGCGTCTCTACCCTCACTCCCTCA |
| G2 | 60bp deletion | GCATCCCTCGGTATCACTCACAGTCGCCCAGCATGTGTGACAGAAAGGAGTTTGTCTTCTCTTTCAATGCCATGGCCTCTTCTTCTATGCATACAACAGGCGGAGGATCTTACTATCACCAGCAGGTCACCTACCAAGACATCAAGCCGTG.~~TGTGATG~~**~~TGA~~**~~GGTGAGGCCACGGGGCCCTCCAGCCCAGCCTGGCCGGCCCAGGGACCAGG~~.AGCCCACCGCCACAAACTGCTTTACTCTGGAGGTATAACCCGTCAGCAAGTGAAAAGGGATAGCCCCACCCCTAACGGATTATTTGTAAAGAAAATCCCAACACAGACTGGGAGCAGCGTCTCTACCCTCACTCCCTCA |

**Supplemental Table 1.** Allele alterations of 3 selected W4 heterozygous clones (C2, E4, G2) from the 30 which were genetically modified using CRISPR/Cas9, compared to wildtype (WT).

**Supplemental Figure 1.** **Generation of novel FOXF1:GFP ESC line using CRISPR/Cas9.** **A)** Schematic shows the targeting strategy for inserting AA-GFP into the endogenous mouse *Foxf1* gene locus to generate the FOXF1:GFP ESC line. PCR sequences used for clone screening are included in the schematic. **B)** PCR gel shows randomly selected clones for screening of the FOXF1:GFP insert. Clone A1 was identified as being homozygous with expected fragment sizes WT: 317bp and knock-in: 162bp. Further analysis confirmed homozygosity or heterozygosity. **C)** Immunofluorescent image and dot plot show baseline GFP expression in undifferentiated A1 ESCs after 9 days in culture with 2i maintenance media. Graph represents quantification of the percentage of endogenous GFP levels in the  A1 cell line compared to the parental W4 cell line.

**Supplemental Figure 2.** **Spontaneous differentiation of A1 ESCs results in FOXF1 expression detected by GFP.** **A)** Immunofluorescent images showing the addition of Vascular endothelial growth factor (VEGF) to ESC maintenance media results in spontaneous differentiation and expression of FOXF:GFP in some A1 cells but not in the parental W4 line under the same conditions. **B)** Immunofluorescent images showing the addition of Fibroblast growth factor 2 (FGF2) to ESC maintenance media results in spontaneous differentiation and expression of FOXF:GFP in some A1 cells but not in the parental W4 line under the same conditions. **C)** Immunofluorescent images showing that no spontaneous differentiation occurs in either A1 or W4 ESC lines with 2i maintenance media alone. Scale bars = 50μm.

**Supplemental Figure 3. Differentiated W4, A1, and endogenous mouse lung endothelial cells reveal similarities in expression of various endothelial markers.** Dot plots show the FACS-gating strategy to identify live endothelial cells (CD31^+^CD45^-^), endothelial progenitor cells (c-KIT^+^FOXF1^+^), and various other endothelial cell markers: Vascular endothelial growth factor receptor 2 (VEGFR2, CD309), CD34, and VE-cadherin (CD144) in **A)** mouse lung tissue **B)** differentiated parental W4 ESCs, and **C)** differentiated A1 FOXF1:GFP ESCs.

**Supplemental Figure 4. Bulk injection of differentiated ESCs results in multicellular integration clusters that are restricted to the pulmonary tissue and express early lung development markers A)** Photos show the lungs of EPC-injected mice at P15 with the red dots being areas of multicellular tdTomato^+^ clusters in the lung. **B)** Immunostaining shows tdTomato^+^ donor cells express lung markers such as NKX2.1 (TTF1), SRY-box transcription factor 2 (SOX2), and SRY-box transcription factor 9 (SOX9). Multicellular integration clusters contain regions of uncompacted cells (indicated by arrows). Scale bars = 50μm. **C)** Immunostaining shows that tdTomato^+^ cell integration is restricted to lung tissue of recipient mice. Scale bars = 100μm, 50μm (insert). **D)** Analysis of bone marrow cells shows no tdTomato+ cells in the bone marrow compartment of EPC-injected mice as measured by mean fluorescent intensity (MFI) and compared to non-injected and tdTomato transgenic mice (positive control).

**Supplemental Figure 5. Multicellular integration resembles early developing lung tissue. A)** Hematoxylin and eosin (H&E) staining shows sections of the pulmonary alveolar region in room air (RA), PBS-injected, and tdTomato^+^ EPC-injected mice. PBS- and ESC-injected mice were injured by hyperoxia from P1-P7. Regions of multicellular integration can be found in tdTomato^+^ EPC-injected sections with areas of uncompacted cells (indicated by arrowheads) and primitive epithelial tubules that closely resemble embryonic lung tissue. **B)** Immunostaining of tdTomato^+^ EPC-injected mice reveal SOX2 expression in areas of forming- and well-formed-tubules (indicated by arrowheads) within the areas of multicellular integration. Scale bars = 50μm.

**Supplemental Figure 6. Characterization of multicellular engraftment reveals expression of endothelial marker, endomucin and non-endothelial markers.** Immunostaining shows that regions of multicellular integration, post-EPC injection, contain expression of **A)** Ki67 **B)** Caspase-3 **C)** Endomucin **D)** alpha-smooth muscle actin (α-SMA). Scale bars = 50μm.

**Supplemental Figure 7. Injection of donor EPCs results in limited single-cell integration in the alveolar region. A)** Fluorescent images show day 5 tdTomato^+^ differentiated EPCs in vitro, prior to harvest and FACS sorting. Scale bar = 50μm. **B)** Workflow shows the experimental design with 100,000 EPCs injected. Immunostaining of lung tissue at P21 revealed minimal single-cell integration of donor tdTomato^+^ EPCs in the distal lung, co-stained with endothelial marker CD31. Scale bars = 50μm. **C)** Fluorescent images of lungs after 400,000 EPC injection and harvest at P21 show that retro orbital injection is effective at delivering donor cells (tdTomato^+^) specifically to lung tissue post-injury. Scale bar = 100μm. **D)** Immunostaining shows a low magnification view of tdTomato^+^ EPC engraftment in the alveolar lung region (P21), after retro orbital injection of 400,000 ESC-derived EPCs at P5. Immunostaining for CD31 shows co-expression of tdTomato, FOXF1:GFP, and CD31 in the vascular network of recipient mice. Scale bar = 50μm.

**Supplemental Figure 8. Gating strategy for identification of various cell populations in the murine lung. A)** Dot plots show the gating strategy used to identify various cell populations in the murine lung across room air, hyperoxia (HO), and hyperoxia with EPC injection groups. Lung tissues were enzymatically digested and stained for CD31 and CD45. Data was acquired using FACSAria II and analyzed using FlowJo software. Cells were gated in a stepwise manner, beginning with all acquired cells, doublet exclusion, dead cell exclusion, and gating of the four major cell groups: Endothelial (CD31^+^CD45^-^), Double positive (CD31^+^CD45^+^), Double negative (CD31^-^CD45^-^), and Hematopoietic (CD31^-^CD45^+^).
