# Supplementary material for: Endothelial progenitor cells derived from embryonic stem cells prevent alveolar simplification in a murine model of bronchopulmonary dysplasia
Source: Front Cell Dev Biol. 2023 Jun 9;11:1209518. doi: 10.3389/fcell.2023.1209518 (PMC10289167; doi:10.3389/fcell.2023.1209518)
Supplement: Supplementary file 3 [file Presentation1.PDF]

SUPPLEMENTAL FIGURE LEGENDS:

Clone	Allele alteration	Sequence
**WT**	None	GCATCCCTCGGTATCACTCACAGTCGCCCAGCATGTGTGACAGAAAGGA<br>GTTTGTCTTCTCTTTCAATGCCATGGCCTCTTCTTCTATGCATACAACAGG<br>CGGAGGATCTTACTATCACCAGCAGGTCACCTACCAAGACATCAAGCCG<br>TGTGTGATG**TGA**GGTGAGGCCACGGGGCCCTCCAGCCCAGCCTGGCCG<br>GCCCAGGGACCAGGAGCCCACCGCCACAAACTGCTTTACTCTGGAGGT<br>ATAACCCGTCAGCAAGTGAAAAGGGATAGCCCCACCCCTAACGGATTAT<br>TTGTAAAGAAAATCCCAACACAGACTGGGAGCAGCGTCTCTACCCTCA<br>CTCCCTCA
**C2**	1bp insertion	GCATCCCTCGGTATCACTCACAGTCGCCCAGCATGTGTGACAGAAAGGA<br>GTTTGTCTTCTCTTTCAATGCCATGGCCTCTTCTTCTATGCATACAACAGG<br>CGGAGGATCTTACTATCACCAGCAGGTCACCTACCAAGACATCAAGCCG<br>TGTGaTGATG**TGA**GGTGAGGCCACGGGGCCCTCCAGCCCAGCCTGGCC<br>GGCCCAGGGACCAGGAGCCCACCGCCACAAACTGCTTTACTCTGGAGG<br>TATAACCCGTCAGCAAGTGAAAAGGGATAGCCCCACCCCTAACGGATTA<br>TTTGTAAAGAAAATCCCAACACAGACTGGGAGCAGCGTCTCTACCCTC<br>ACTCCCTCA
**E4**	5bp deletion	GCATCCCTCGGTATCACTCACAGTCGCCCAGCATGTGTGACAGAAAGGA<br>GTTTGTCTTCTCTTTCAATGCCATGGCCTCTTCTTCTATGCATACAACAGG<br>CGGAGGATCTTACTATCACCAGCAGGTCACCTACCAAGACATCAAG~~C.C~~<br>~~GTGT~~.GTGATG**TGA**GGTGAGGCCACGGGGCCCTCCAGCCCAGCCTGGCC<br>GGCCCAGGGACCAGGAGCCCACCGCCACAAACTGCTTTACTCTGGAGG<br>TATAACCCGTCAGCAAGTGAAAAGGGATAGCCCCACCCCTAACGGATTA<br>TTTGTAAAGAAAATCCCAACACAGACTGGGAGCAGCGTCTCTACCCTC<br>ACTCCCTCA
**G2**	60bp deletion	GCATCCCTCGGTATCACTCACAGTCGCCCAGCATGTGTGACAGAAAGGA<br>GTTTGTCTTCTCTTTCAATGCCATGGCCTCTTCTTCTATGCATACAACAGG<br>CGGAGGATCTTACTATCACCAGCAGGTCACCTACCAAGACATCAAGCCG<br>TG.~~TGTGATG**TGA**GGTGAGGCCACGGGGCCCTCCAGCCCAGCCTGGCCG~~<br>~~GCCCAGGGACCAGG~~.AGCCCACCGCCACAAACTGCTTTACTCTGGAGGT<br>ATAACCCGTCAGCAAGTGAAAAGGGATAGCCCCACCCCTAACGGATTAT<br>TTGTAAAGAAAATCCCAACACAGACTGGGAGCAGCGTCTCTACCCTCA<br>CTCCCTCA

Supplemental Table 1. Allele alterations of 3 selected W4 heterozygous clones (C2, E4, G2) from the 30 which were genetically modified using CRISPR/Cas9, compared to wildtype (WT).

Supplemental Figure 1. Generation of novel FOXF1:GFP ESC line using CRISPR/Cas9. A) Schematic shows the targeting strategy for inserting AA-GFP into the endogenous mouse *Foxf1* gene locus to generate the FOXF1:GFP ESC line. PCR sequences used for clone screening are included in the schematic. **B)** PCR gel shows randomly selected clones for screening of the FOXF1:GFP insert. Clone A1 was identified as being homozygous with expected fragment sizes WT: 317bp and knock-in: 162bp. Further analysis confirmed homozygosity or heterozygosity. **C)** Immunofluorescent image and dot plot show baseline GFP expression in undifferentiated A1 ESCs after 9 days in culture with 2i maintenance media. Graph represents quantification of the percentage of endogenous GFP levels in the A1 cell line compared to the parental W4 cell line.

Supplemental Figure 2. Spontaneous differentiation of A1 ESCs results in FOXF1 expression detected by GFP. A) Immunofluorescent images showing the addition of Vascular endothelial growth factor (VEGF) to ESC maintenance media results in spontaneous differentiation and expression of FOXF:GFP in some A1 cells but not in the parental W4 line under the same conditions. **B)** Immunofluorescent images showing the addition of Fibroblast growth factor 2 (FGF2) to ESC maintenance media results in spontaneous differentiation and expression of FOXF:GFP in some A1 cells but not in the parental W4 line under the same conditions. **C)** Immunofluorescent images showing that no spontaneous differentiation occurs in either A1 or W4 ESC lines with 2i maintenance media alone. Scale bars = 50μm.

Supplemental Figure 3. Differentiated W4, A1, and endogenous mouse lung endothelial cells reveal similarities in expression of various endothelial markers. Dot plots show the FACS-gating strategy to identify live endothelial cells ($CD31^{+}CD45^{-}$), endothelial progenitor cells (c-$KIT^{+}FOXF1^{+}$), and various other endothelial cell markers: Vascular endothelial growth factor receptor 2 (VEGFR2, CD309), CD34, and VE-cadherin (CD144) in **A)** mouse lung tissue **B)** differentiated parental W4 ESCs, and **C)** differentiated A1 FOXF1:GFP ESCs.

Supplemental Figure 4. Bulk injection of differentiated ESCs results in multicellular integration clusters that are restricted to the pulmonary tissue and express early lung development markers A) Photos show the lungs of EPC-injected mice at P15 with the red dots being areas of multicellular tdTomato^{+} clusters in the lung. **B)** Immunostaining shows tdTomato^{+} donor cells express lung markers such as NKX2.1 (TTF1), SRY-box transcription factor 2 (SOX2), and SRY-box transcription factor 9 (SOX9). Multicellular integration clusters contain regions of uncompacted cells (indicated by arrows). Scale bars = 50μm. **C)** Immunostaining shows that tdTomato^{+} cell integration is restricted to lung tissue of recipient mice. Scale bars = 100μm, 50μm (insert). **D)** Analysis of bone marrow cells shows no tdTomato+ cells in the bone marrow compartment of EPC-injected mice as measured by mean fluorescent intensity (MFI) and compared to non-injected and tdTomato transgenic mice (positive control).

Supplemental Figure 5. Multicellular integration resembles early developing lung tissue. A) Hematoxylin and eosin (H&E) staining shows sections of the pulmonary alveolar region in room air (RA), PBS-injected, and tdTomato^{+} EPC-injected mice. PBS- and ESC-injected mice were injured by hyperoxia from P1-P7. Regions of multicellular integration can be found in tdTomato^{+} EPC-injected sections with areas of uncompacted cells (indicated by arrowheads) and primitive epithelial tubules that closely resemble embryonic lung tissue. **B)** Immunostaining of tdTomato^{+}

EPC-injected mice reveal SOX2 expression in areas of forming- and well-formed-tubules (indicated by arrowheads) within the areas of multicellular integration. Scale bars = 50μm.

Supplemental Figure 6. Characterization of multicellular engraftment reveals expression of endothelial marker, endomucin and non-endothelial markers. Immunostaining shows that regions of multicellular integration, post-EPC injection, contain expression of **A)** Ki67 **B)** Caspase-3 **C)** Endomucin **D)** alpha-smooth muscle actin (α-SMA). Scale bars = 50μm.

Supplemental Figure 7. Injection of donor EPCs results in limited single-cell integration in the alveolar region. A) Fluorescent images show day 5 $tdTomato^+$ differentiated EPCs in vitro, prior to harvest and FACS sorting. Scale bar = 50μm. **B)** Workflow shows the experimental design with 100,000 EPCs injected. Immunostaining of lung tissue at P21 revealed minimal single-cell integration of donor $tdTomato^+$ EPCs in the distal lung, co-stained with endothelial marker CD31. Scale bars = 50μm. **C)** Fluorescent images of lungs after 400,000 EPC injection and harvest at P21 show that retro orbital injection is effective at delivering donor cells ($tdTomato^+$) specifically to lung tissue post-injury. Scale bar = 100μm. **D)** Immunostaining shows a low magnification view of $tdTomato^+$ EPC engraftment in the alveolar lung region (P21), after retro orbital injection of 400,000 ESC-derived EPCs at P5. Immunostaining for CD31 shows co-expression of tdTomato, FOXF1:GFP, and CD31 in the vascular network of recipient mice. Scale bar = 50μm.

Supplemental Figure 8. Gating strategy for identification of various cell populations in the murine lung. A) Dot plots show the gating strategy used to identify various cell populations in the murine lung across room air, hyperoxia (HO), and hyperoxia with EPC injection groups. Lung tissues were enzymatically digested and stained for CD31 and CD45. Data was acquired using FACSAria II and analyzed using FlwoJo software. Cells were gated in a stepwise manner, beginning with all acquired cells, doublet exclusion, dead cell exclusion, and gating of the four major cell groups: Endothelial ($CD31^+CD45^-$), Double positive ($CD31^+CD45^+$), Double negative ($CD31^-CD45^-$), and Hematopoietic ($CD31^-CD45^+$).

A

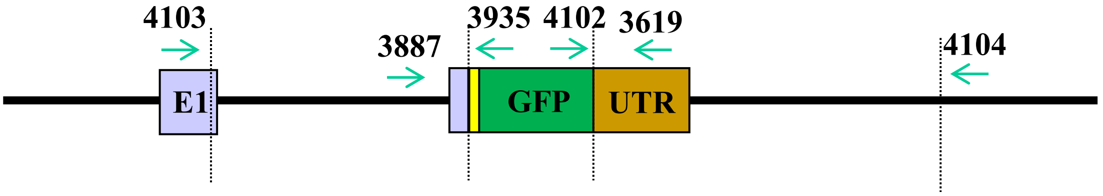

VS3619: AGGGAGTGAGGGTAGAGACG
VS3887: CATGTGTGACAGAAAGGAGTTTGTC
VS3935: TGCTTCAGCAGGCTGAAGTTAGTAGC
VS4102: CACTCTCGGCATGGACGAGCTGTAC
VS4103: TGGAGCAGCCATACCTTCACCAAAAC
VS4104: TGCCTACGTGCTCCCCTTGTTTTCAC

B

Internal PCR:

	WT	KI
3887/3619/3935:	317 bp	162 bp
3887/3619/4102:	317 bp	233 bp

External PCR:

	WT	KI
4103/3935:	-	1591 bp
4102/4104:	-	2526 bp

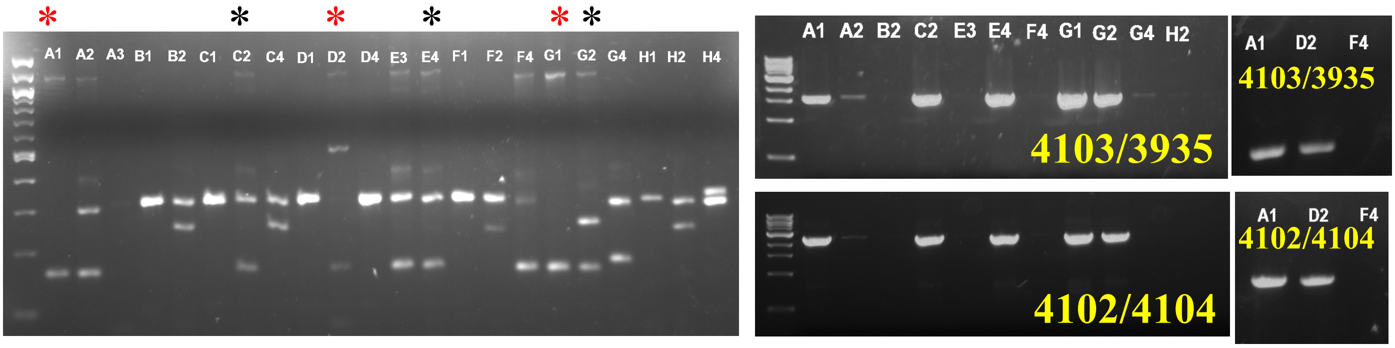

C

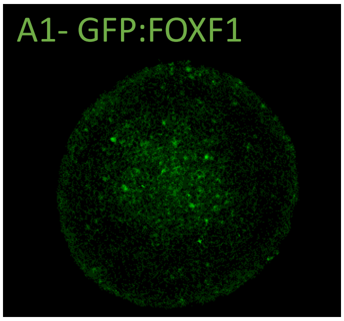

Undifferentiated- A1

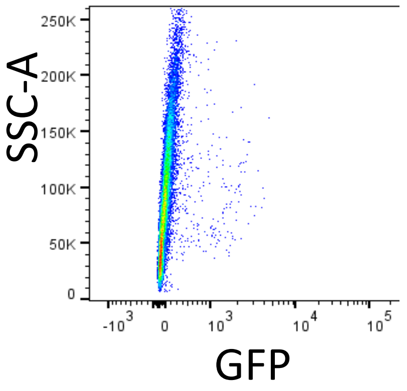

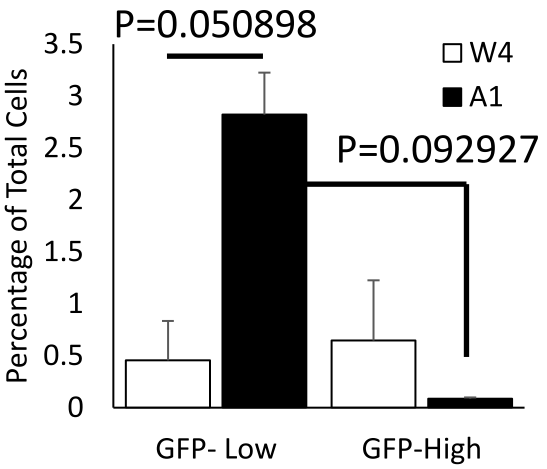

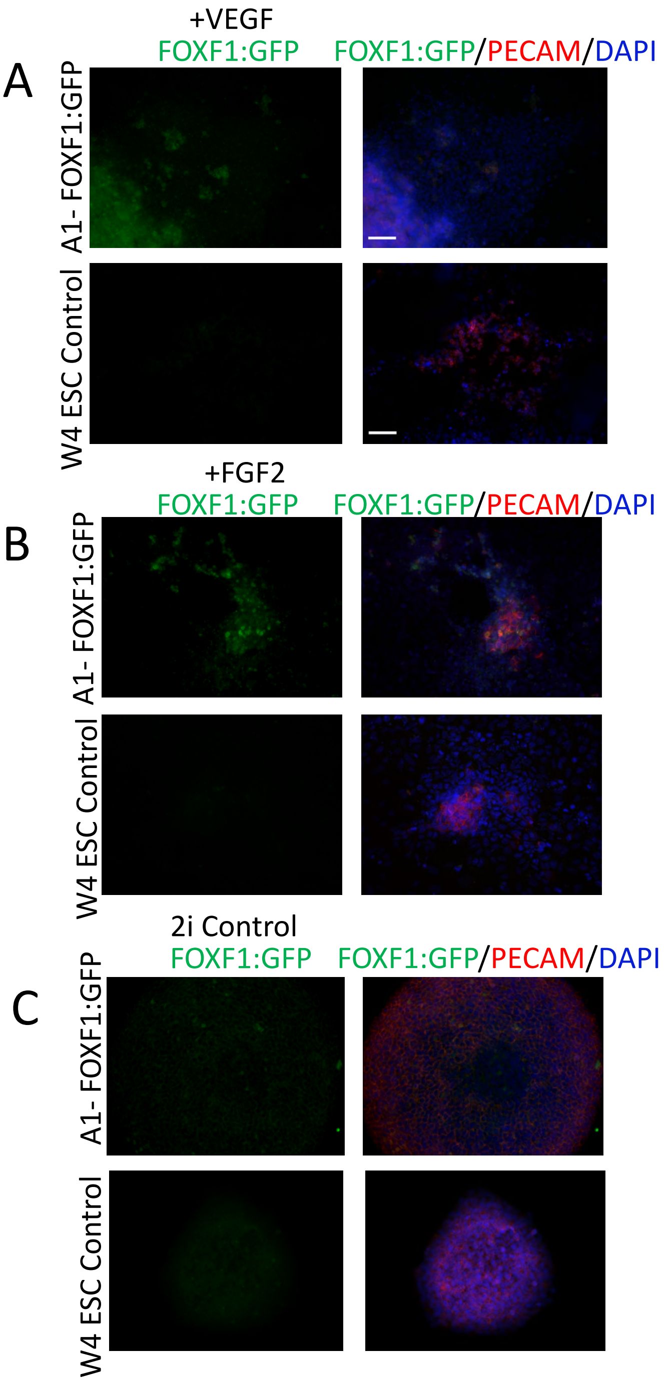
+VEGF
FOXF1:GFP
FOXF1:GFP/PECAM/DAPI
A
A1- FOXF1:GFP
W4 ESC Control
+FGF2
FOXF1:GFP
FOXF1:GFP/PECAM/DAPI
B
A1- FOXF1:GFP
W4 ESC Control
2i Control
FOXF1:GFP
FOXF1:GFP/PECAM/DAPI
C
A1- FOXF1:GFP
W4 ESC Control

Kolesnichenko et al Suppl. Fig.3

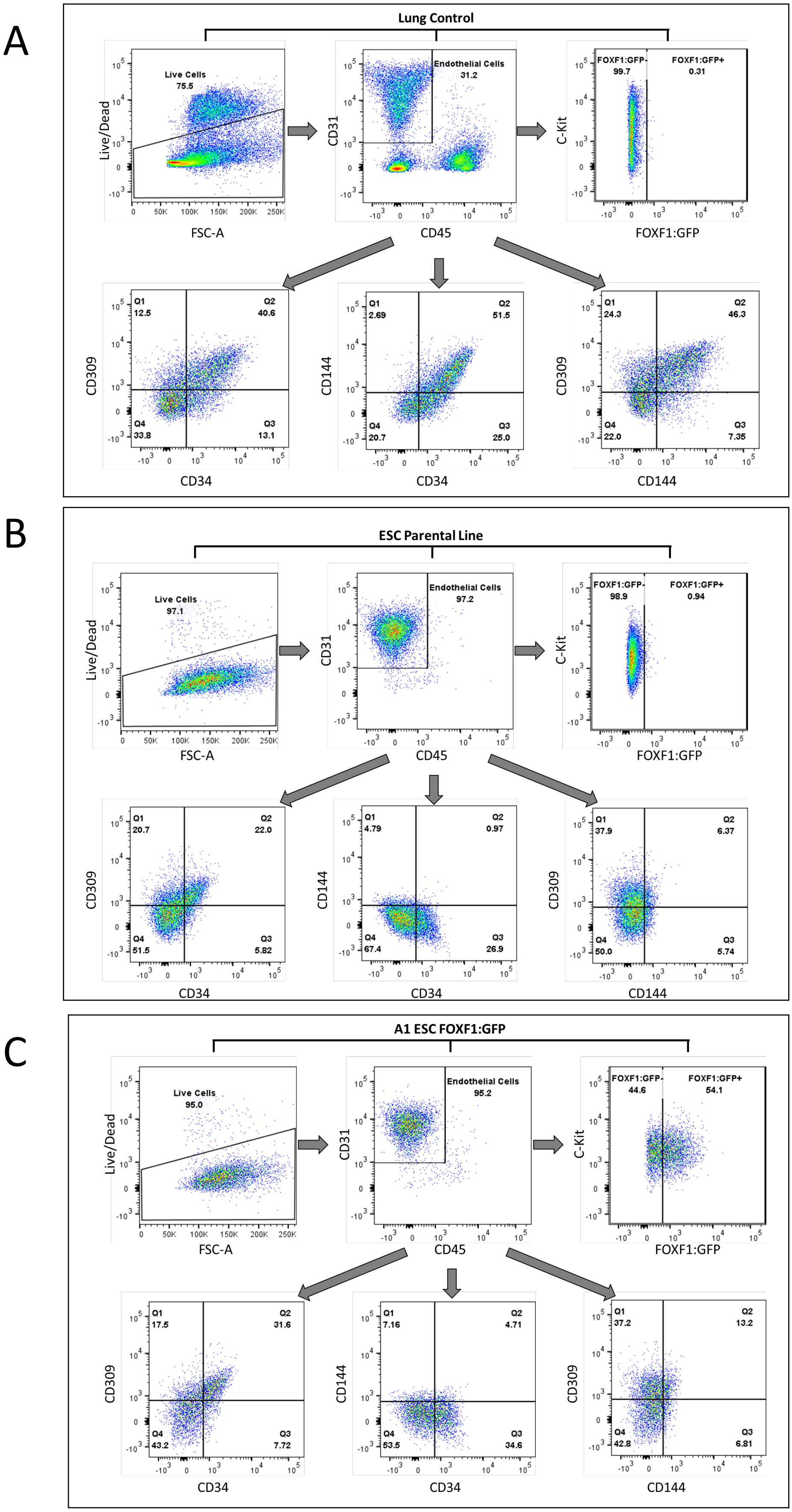

Kolesnichenko et al Suppl. Fig.4

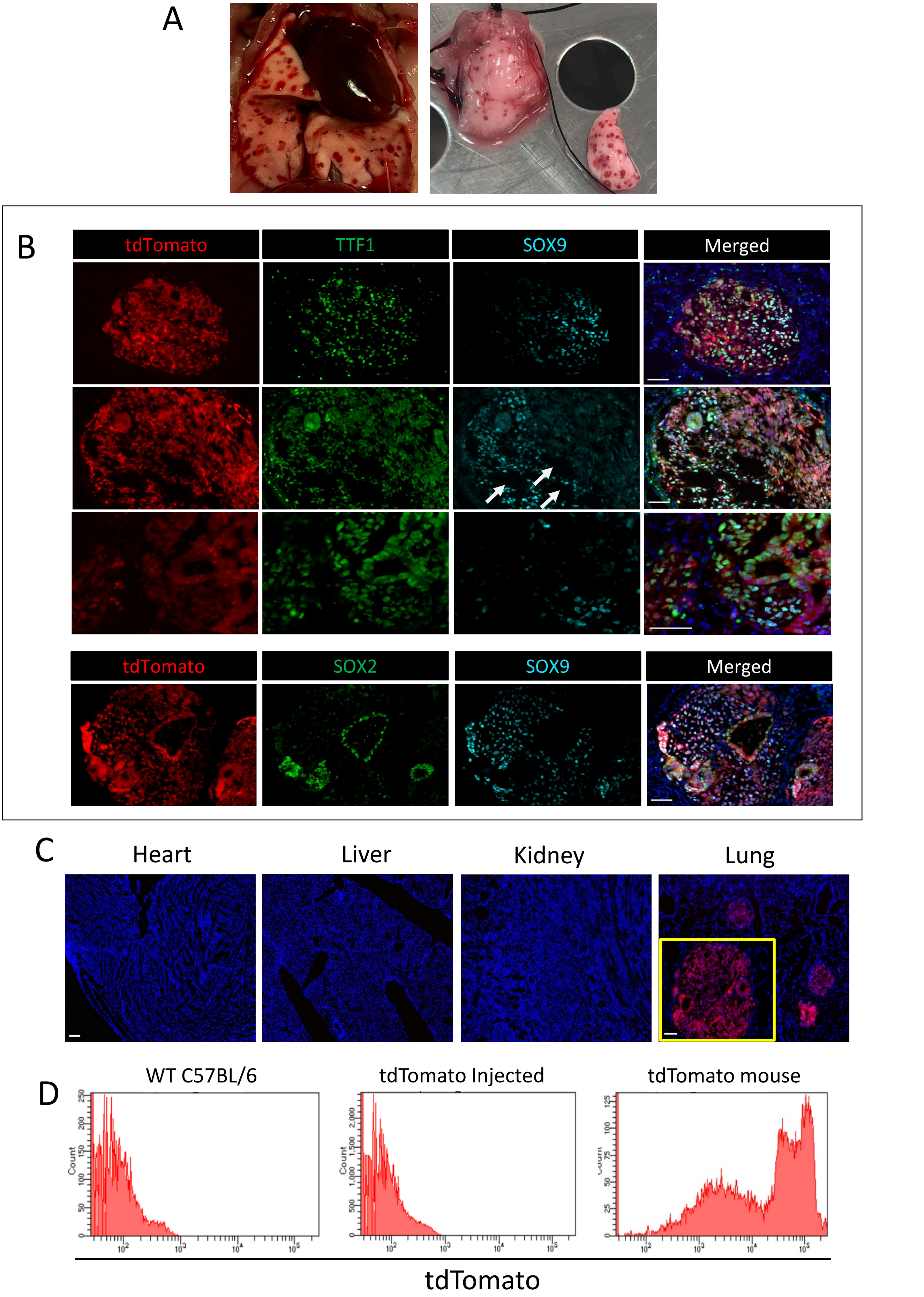

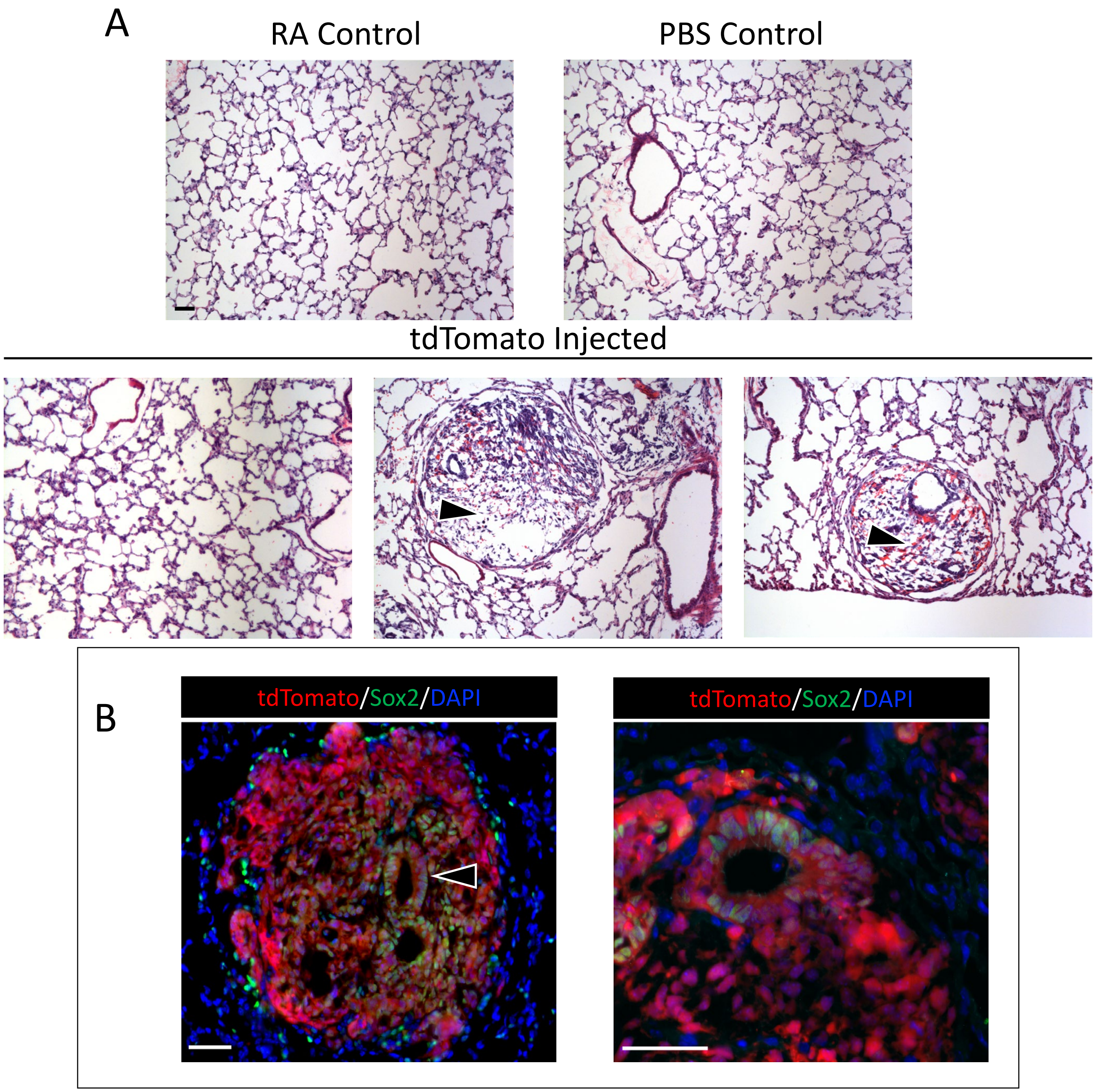
A
RA Control
PBS Control
tdTomato Injected
B
tdTomato/Sox2/DAPI
tdTomato/Sox2/DAPI

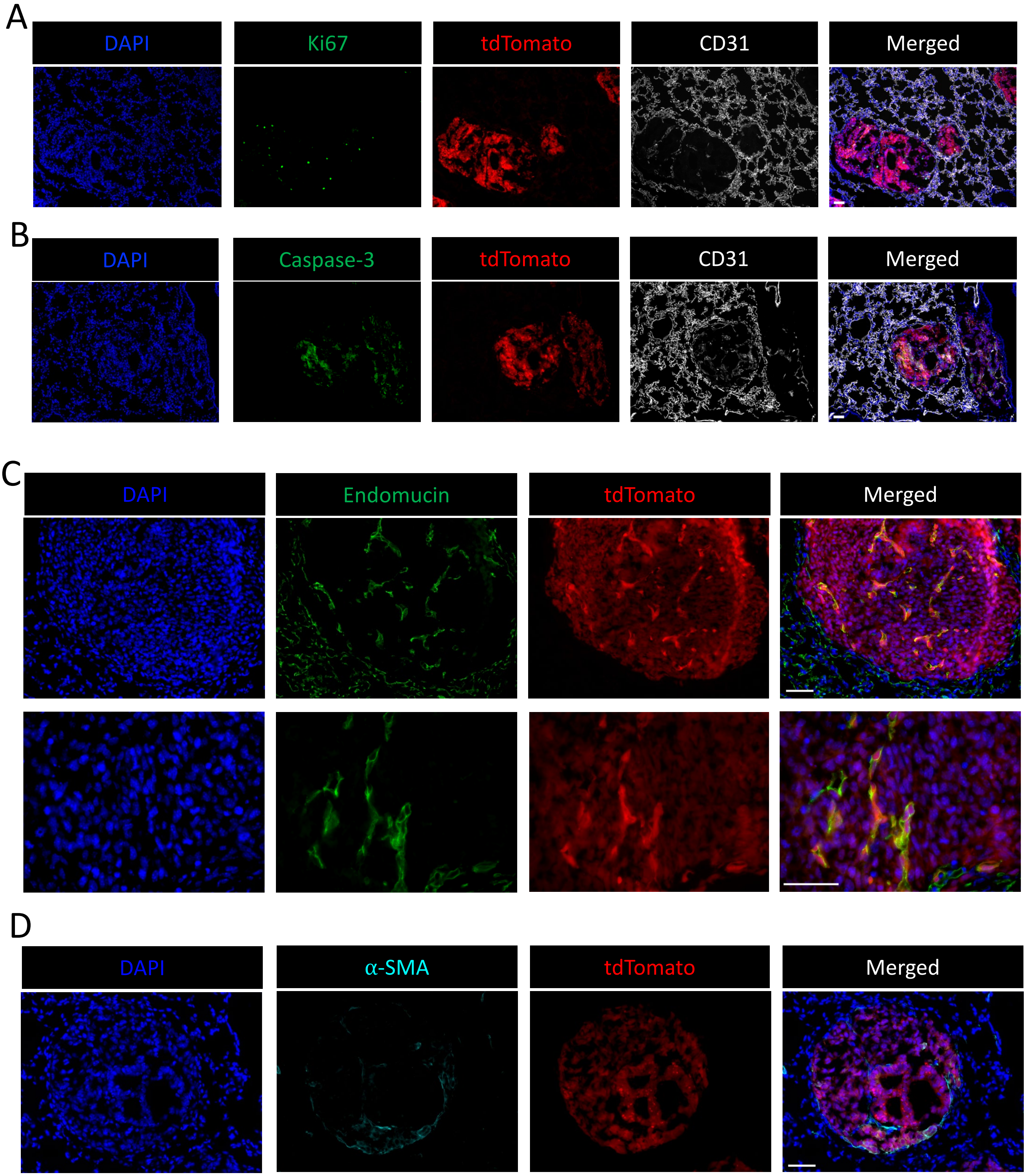
A
DAPI
Ki67
tdTomato
CD31
Merged
B
DAPI
Caspase-3
tdTomato
CD31
Merged
C
DAPI
Endomucin
tdTomato
Merged
D
DAPI
α-SMA
tdTomato
Merged

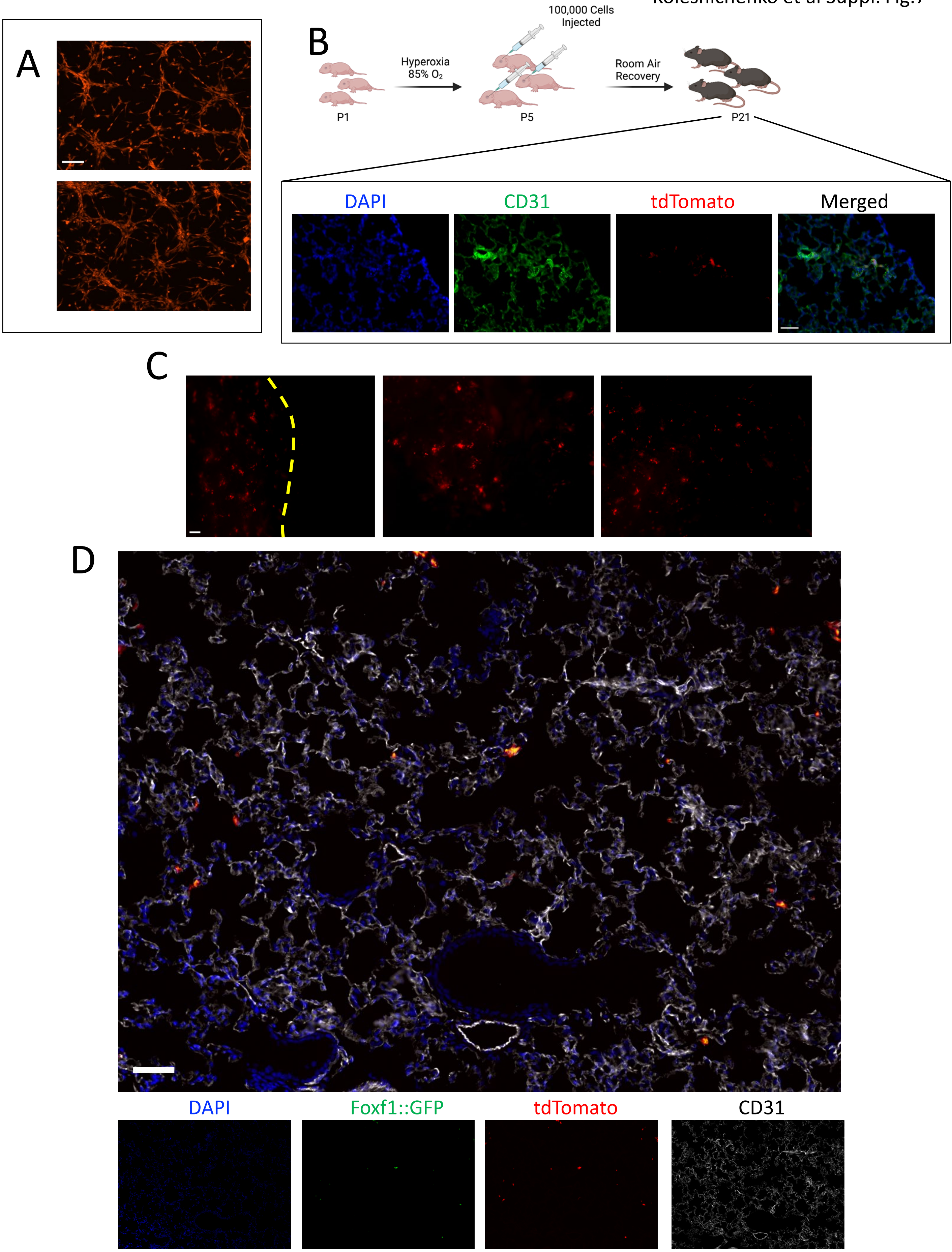
A
B
100,000 Cells
Injected
Hyperoxia
85% O2
Room Air
Recovery
P1
P5
P21
DAPI
CD31
tdTomato
Merged
C
D
DAPI
Foxf1::GFP
tdTomato
CD31

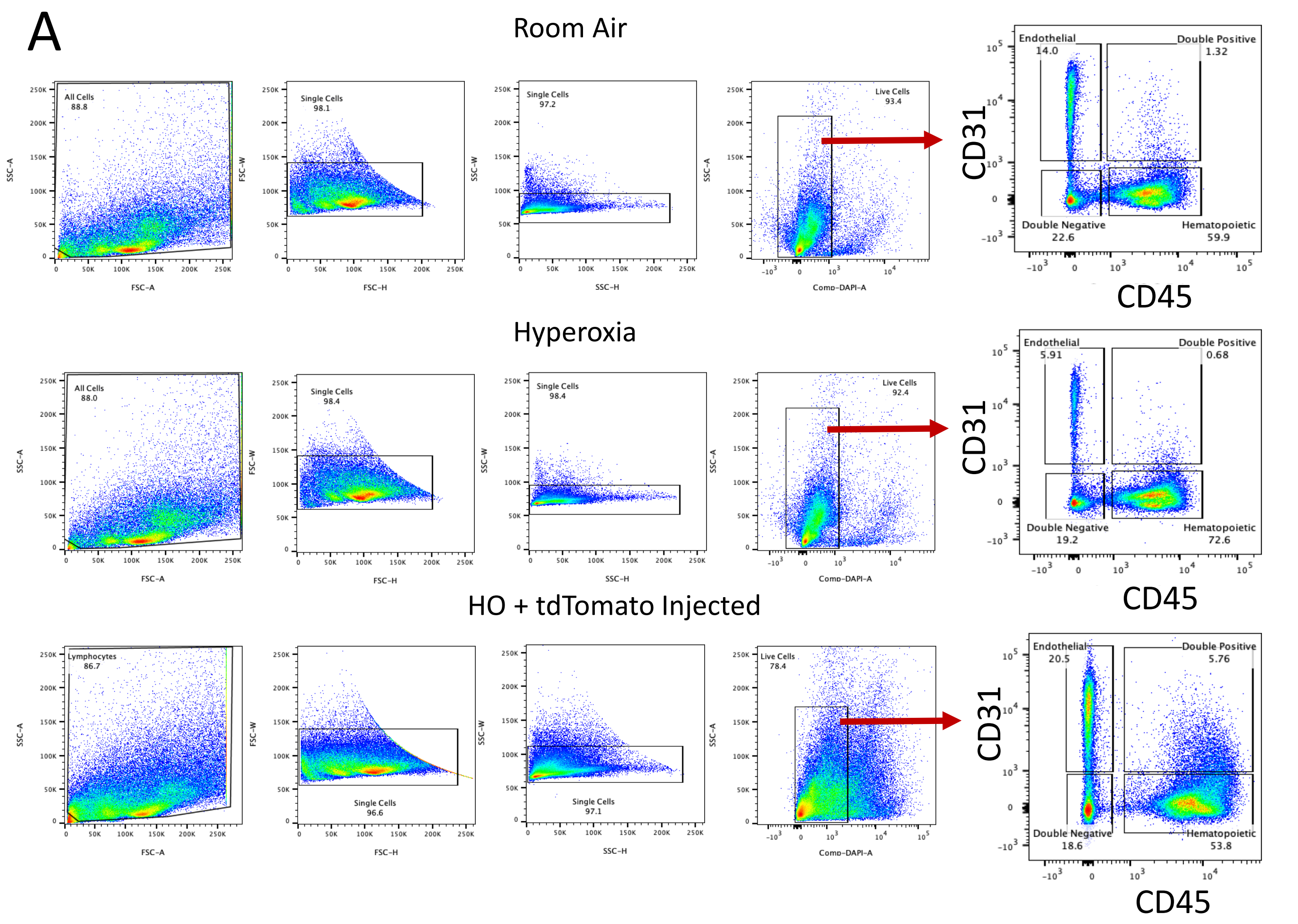
A
Room Air
All Cells
88.8
Single Cells
98.1
Single Cells
97.2
Live Cells
93.4
Endothelial
14.0
Double Positive
1.32
Double Negative
22.6
Hematopoietic
59.9
CD31
CD45
Hyperoxia
All Cells
88.0
Single Cells
98.4
Single Cells
98.4
Live Cells
92.4
Endothelial
5.91
Double Positive
0.68
Double Negative
19.2
Hematopoietic
72.6
CD31
CD45
HO + tdTomato Injected
Lymphocytes
86.7
Single Cells
96.6
Single Cells
97.1
Live Cells
78.4
Endothelial
20.5
Double Positive
5.76
Double Negative
18.6
Hematopoietic
53.8
CD31
CD45
SSC-A
FSC-A
FSC-W
FSC-H
SSC-W
SSC-H
Comp-DAPI-A
